# The antiviral response triggered by the cGAS/STING pathway is subverted by the foot-and-mouth disease virus proteases

**DOI:** 10.1007/s00018-024-05190-7

**Published:** 2024-03-20

**Authors:** Miguel Ángel Sanz, Miryam Polo, Miguel Rodríguez-Pulido, Ranjitha Huildore Bommanna, Margarita Sáiz

**Affiliations:** Centro de Biología Molecular Severo Ochoa, CSIC-UAM, Madrid, Spain

**Keywords:** cGAS, Foot-and-mouth disease virus, Antiviral immunity, Immune evasion, Leader protease, 3C protease

## Abstract

Propagation of viruses requires interaction with host factors in infected cells and repression of innate immune responses triggered by the host viral sensors. Cytosolic DNA sensing pathway of cyclic GMP-AMP synthase (cGAS) and stimulator of interferon genes (STING) is a major component of the antiviral response to DNA viruses, also known to play a relevant role in response to infection by RNA viruses, including foot-and-mouth disease virus (FMDV). Here, we provide supporting evidence of cGAS degradation in swine cells during FMDV infection and show that the two virally encoded proteases, Leader (Lpro) and 3Cpro, target cGAS for cleavage to dampen the cGAS/STING-dependent antiviral response. The specific target sequence sites on swine cGAS were identified as Q140/T141 for the FMDV 3Cpro and the KVKNNLKRQ motif at residues 322–330 for Lpro. Treatment of swine cells with inhibitors of the cGAS/STING pathway or depletion of cGAS promoted viral infection, while overexpression of a mutant cGAS defective for cGAMP synthesis, unlike wild type cGAS, failed to reduce FMDV replication. Our findings reveal a new mechanism of RNA viral antagonism of the cGAS-STING innate immune sensing pathway, based on the redundant degradation of cGAS through the concomitant proteolytic activities of two proteases encoded by an RNA virus, further proving the key role of cGAS in restricting FMDV infection.

**Supplementary Information:**

The online version contains supplementary material available at 10.1007/s00018-024-05190-7.

## Introduction

The innate immune response is the first line of defense against viral infection in mammalian cells. Although infection by RNA viruses is mainly detected by RNA sensing pattern recognition receptors (PRRs) including toll‐like receptors (TLRs) and retinoic acid inducible gene I (RIG‐I)‐like receptors (RLRs) [1–3], recent research highlights the importance of DNA sensors in RNA virus biology and disease pathogenesis [4]. Specifically, the cyclic GMP‐AMP synthase (cGAS)‐ stimulator of interferon genes (STING) signaling axis has been shown to play an essential role in fighting infections by RNA viruses but also contributing to excessive inflammation associated with viral pathogenesis [5]. Upon detection and binding of pathogen-derived or mislocalized DNA, cGAS catalyzes the synthesis of the second messenger 2′-3′-cyclic GMP-AMP (cGAMP), which then binds to STING inducing its translocation from the ER to the Golgi for activation by post-translational modifications [6]. Activated STING induces the activation of the TBK1 kinase and phosphorylation of IRF3 promoting the transcription of type-I interferon (IFN-I) [7]*.*

Foot-and-mouth disease virus (FMDV) is the causative agent of a highly infectious disease affecting farm and wild animals worldwide with devastating economic impact [8, 9]. FMDV belongs to the *Picornaviridae* family including small non-enveloped positive-strand RNA viruses of significant disease burden for humans and livestock. FMDV is known to evade the host immune response by a number of different strategies mainly orchestrated by the catalytic activity of the two genome encoded proteases [10, 11]. The papain-like cysteine Leader protease (Lpro) is expressed as two forms, being Lbpro the most abundant in FMDV-infected cells [12]. The proteolytic activity of Lpro is implicated in suppressing IFN-α/β induction and a number of host proteins, including the immune RNA sensors LGP2 and MDA5, have been identified as Lpro targets [13, 14]. The FMDV 3Cpro is a trypsin-like serine protease responsible for processing of the viral genome but also involved in degradation of host factors engaged in antiviral response in infected cells like NEMO, TANK or G3BP1 among others [11, 15, 16]. Lpro and 3Cpro both cleave eIF4G at different sites impairing cap-dependent translation leading to host shut-off [17, 18].

Several viral families including DNA and RNA viruses are restricted by the cGAS-STING dependent IFN-I induction and in turn have evolved specific inhibitory mechanisms. These strategies are mainly based on abrogating the enzymatic activity of cGAS [19, 20], inactivating STING [21, 22], or degrading the messenger cGAMP [23]. Among the specific mechanisms developed by RNA viruses [24], it has been described that a direct interaction of the Dengue virus (DENV) NS2B protein with cGAS promotes its lysosomal degradation [25]. Similarly, the CHIKV capsid protein promotes the cGAS autophagy-mediated degradation [26] and also the NS1 protein of Zika virus mediates the stabilization of caspase-1 promoting cGAS degradation [27]. Moreover, the NS1 protein of Influenza A virus has been shown to bind mitochondrial DNA (mtDNA) released to the cytoplasm during infection to evade the cGAS/STING-dependent antiviral immunity [28].

Using recombinant proteins and in vitro cleavage assays, Bhattacharya et al. showed that the DENV NS2B3 protease is capable of cleaving human cGAS in the N-terminus potentially disrupting the C-terminal enzymatic activity of the protein [29]. Also, a recent work showed that the 3C protease encoded by the Seneca valley virus (SVV) can cleave porcine cGAS overexpressed in human cells [30]. Moreover, triggering of IFN-I responses has been associated to mitochondrial damage and DNA release during picornaviral infection and overexpression of the FMDV non-structural 2B protein induced mtDNA release to the cytosol in swine PK-15 cells [31]. Here, we show that endogenous cGAS is degraded during FMDV infection in swine cells and describe the double cleavage of cGAS exerted by the two FMDV-encoded Leader and 3C proteases. The target sites on swine cGAS have been identified for both proteases and the impact of cleavage on the IFN-I-dependent antiviral response analyzed. The effect on FMDV infection of treatment with inhibitors of the cGAS/STING signaling has also been studied and our results support the relevance of this DNA sensing pathway restricting infection by FMDV which in turn has evolved a reinforced counteracting strategy.

## Results

### cGAS is degraded during FMDV infection

**Fig. 1:**
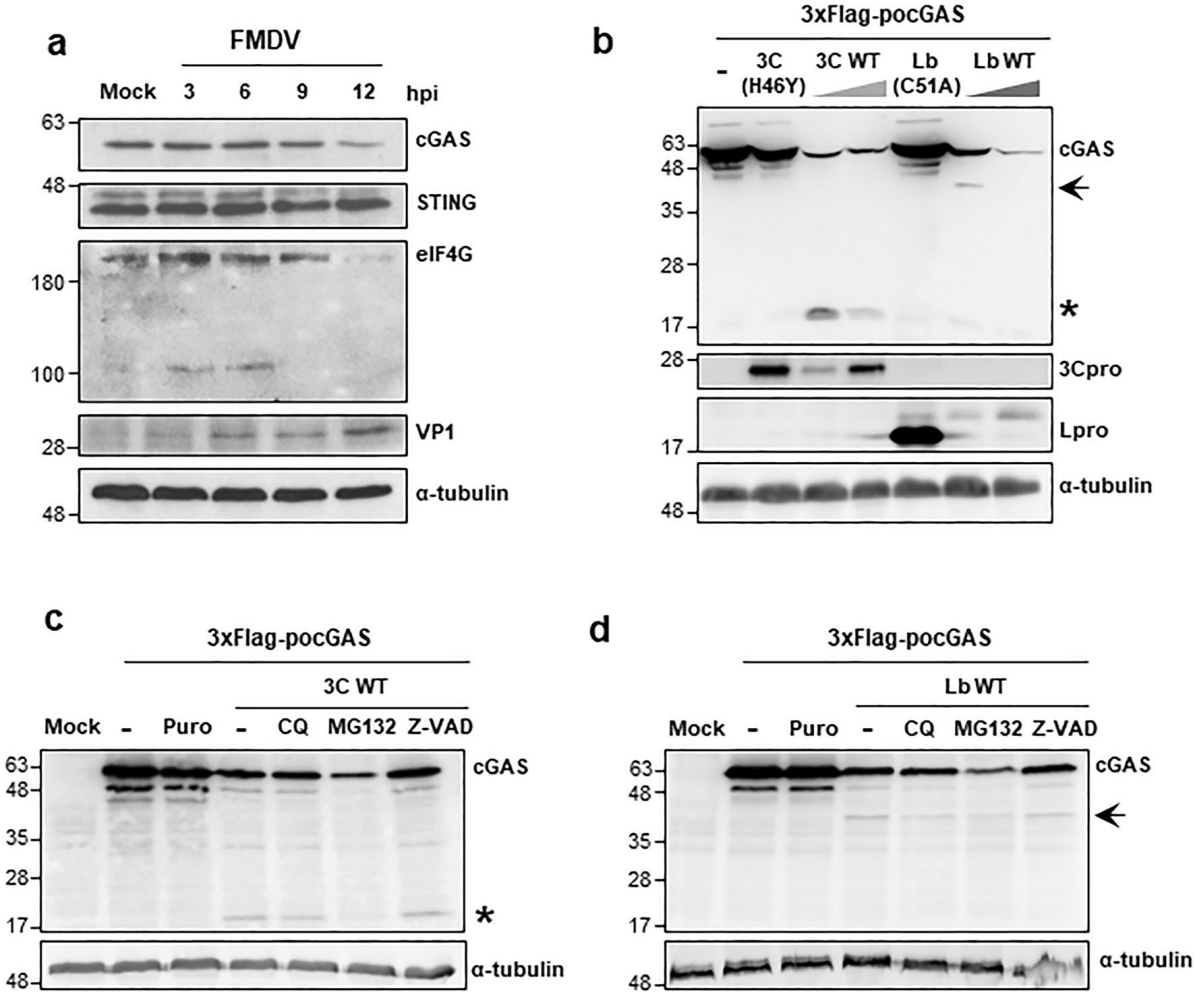
cGAS is degraded during FMDV infection and cleaved by overexpression of 3Cpro or Lbpro. **a** WSL cells were infected with FMDV O1K at an MOI of 1 or mock-infected and lysed at different times after infection for western blot analysis for the indicated proteins. **b** HEK293 cells (2 × 10^5^) were transfected with 3xFlag-*po*cGAS (0.6 µg) or co-transfected with 3xFlag-*po*cGAS (0.6 µg) and either Lbwt or 3Cwt proteases (20 or 50 ng) or their respective catalytically inactive mutants Lb(C51A) and 3C(H46Y) (100 ng). Cells were lysed 24 h after transfection and analysed by western blot. **c**, **d** HEK293 cells (2 × 10^5^) were mock-transfected, transfected with 3xFlag-*po*cGAS (0.6 µg) in the presence or absence of puromycin (20 µM) or co-transfected with 3xFlag-*po*cGAS (0.6 µg) and 20 ng of 3Cwt (**c**) or Lbwt (**d**) in the presence or absence of zVAD-FMK (20 µM), MG132 (10 µM) or CQ (50 µM). Cells were collected 24 h later for western blot analysis for the indicated proteins. The cleavage products generated by 3C and Lb proteases are indicated with an asterisk and with an arrow, respectively

To analyze the integrity of cGAS and STING during FMDV infection, wild boar lung WSL cells were infected with FMDV at a multiplicity of infection (MOI) of 1 (1 pfu/cell) and lysed at different times after infection for western blot analysis (Fig. 1a). A clear decrease in endogenous cGAS levels could be observed at 12 h after infection while STING levels remained more stable. Interestingly, the reduction of cGAS levels correlated with the accumulation of viral protein VP1 and the decay of eIF4GI resulting from the catalytic activity of Lpro [32]. To confirm these results, cGAS and STING levels were analyzed over infection with a higher viral dose (MOI of 5) in swine kidney SK6 cells or with a different FMDV isolate (O1BFS) in WSL cells (Supplementary Fig. 1a, b). Although detection of cGAS in SK6 cells was weak, a trend to reduction was observed at early times of infection while STING and tubulin seemed to follow a similar rate of decay (Supplementary Fig. 1a). Comparison of the effect of the FMDV dose showed an earlier reduction in cGAS levels barely detectable at 9 h after infection. A milder decrease was observed for STING levels still abundant after 12 h of infection (Supplementary Fig. 1b). Although it has been recently reported that FMDV infection induced the decrease of STING but did not affect the expression of cGAS in swine PK15 cells [31], our data suggest that cGAS could be specifically targeted for degradation during FMDV infection. To test that hypothesis, we first studied the effect of co-expression in HEK293 cells of N-terminal FLAG-tagged porcine cGAS (3xFLAG-*po*cGAS) together with the FMDV-encoded proteases Lbpro and 3Cpro either in their catalytically active wild-type forms or their inactive counterparts Lb(C51A) [33] and 3C(H46Y) [34] carrying mutations in their corresponding active sites. As shown in Fig. 1b, co-expression with 3C(H46Y) or Lb(C51A) did not affect *po*cGAS levels, while the co-expression with increasing amounts of catalytically active 3Cpro or Lpro resulted in a strong decrease in the *po*cGAS levels, likely due in part to the proteolytic activity exerted on the protein and also to the translation inhibition effect caused by both proteases [35]. Indeed, in co-expression with 3Cwt a *po*cGAS-derived fragment of approximately 18 kDa was recognized by the anti-tag antibody and a different fragment of about 40 kDa could be detected in co-expression with Lbwt (Fig. 1b). These results suggest that the N-terminal fragments of *po*cGAS detected in co-expression with FMDV 3Cwt and Lbwt may result from specific proteolytic cleavage by both proteases.

To address whether the caspase, proteasomal or lysosomal pathways might be involved in the cGAS degradation pattern observed, we performed the co-expression experiments in the presence of their corresponding specific inhibitors Z-VAD, MG-132 or chloroquine (CQ), respectively. As shown in Fig. 1c, d, both the 18-kDa and 40-kDa *po*cGAS fragments generated in co-expression with 3Cpro and Lbpro, respectively were still detected in the presence of the inhibitors. Also, treatment with puromycin to induce apoptosis did not result in cGAS cleavage. Together, these results suggest that the cGAS degradation products observed were attributable to the specific activities of the FMDV 3C and Lb proteases by direct cleavage. However, the cellular processes of protein degradation triggered by infection may also contribute to the decay observed for both cGAS and STING at late times after infection with high viral doses.

### Identification of the cleavage sites of the FMDV 3Cpro and Lbpro on cGAS

**Fig. 2:**
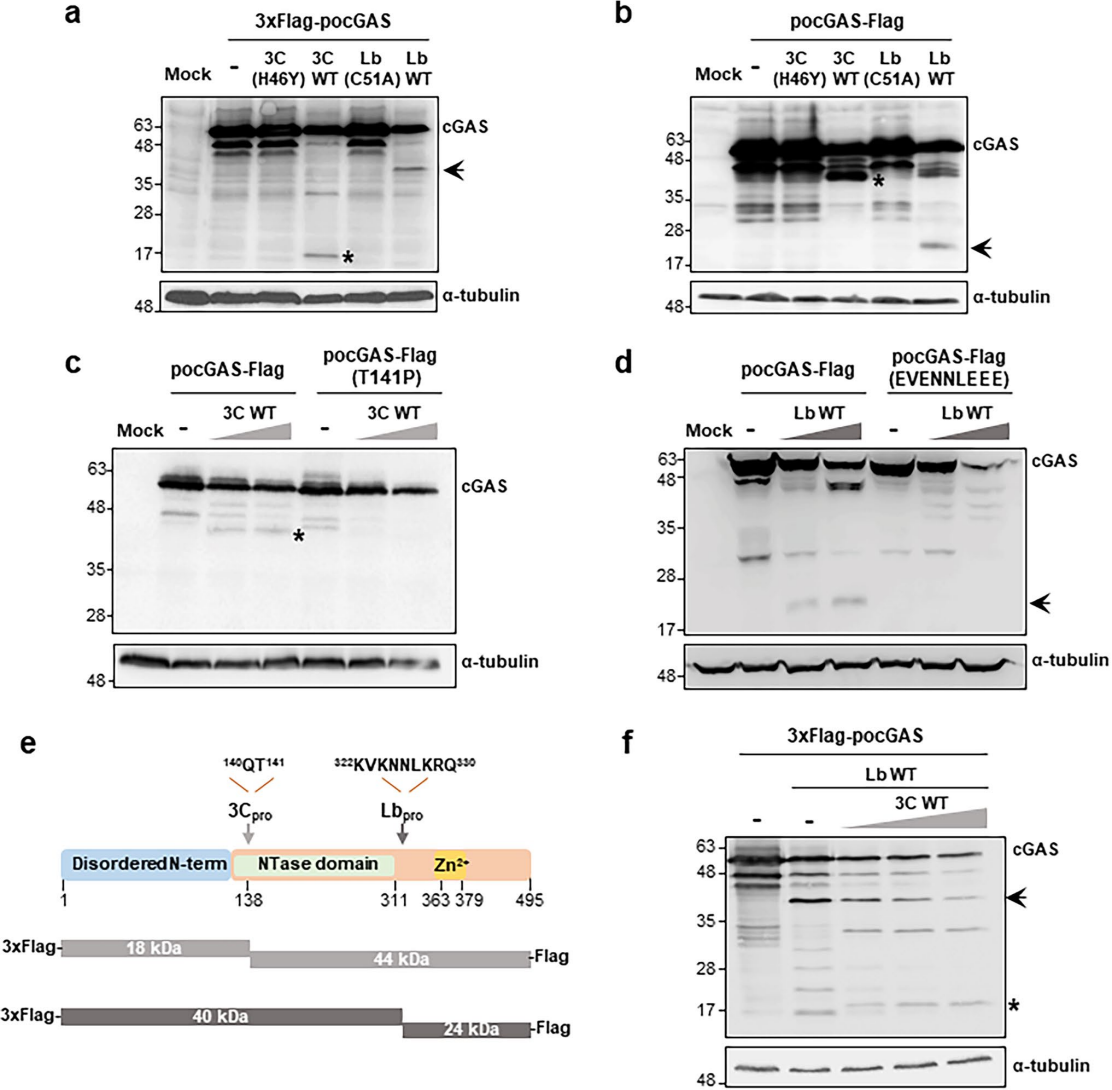
The FMDV proteases cleave *po*cGAS at specific sites. **a**, **b** HEK293 cells (2 × 10^5^) were mock-transfected, transfected with 0.6 µg of 3xFlag-*po*cGAS (**a**) or *po*cGAS-Flag (**b**) or co-transfected with 3C(H46Y) (100 ng), 3Cwt (20 ng), Lb(C51A) (100 ng) or Lbwt (20 ng). **c**, **d** HEK293 cells were co-transfected as above with either 0.6 µg of 3xFlag-*po*cGAS or 3xFlag-*po*cGAS T141P (**c**) or 0.6 µg of 3xFlag-*po*cGAS or 3xFlag-*po*cGAS (KVKNNLKRQ/EVENNLEEE) (**d**) and 0, 20 or 50 ng of 3Cwt (**c**) or Lbwt (**d**), respectively. Cells were lysed 24 h after transfection for western blot analysis for the indicated proteins. The cleavage products generated by 3C and Lb proteases are indicated with an asterisk and with an arrow, respectively. (**e**) Schematic representation showing the cleavage products generated by Lpro and 3Cpro on the *po*cGAS constructs assayed and detected with the anti-Flag antibodies. The different domains and positions are depicted by analogy with the human cGAS sequence. (**f**) HEK293 cells were transfected with 0.6 µg of 3xFlag-*po*cGAS or co-transfected with 3xFlag-*po*cGAS (0.6 µg), Lbwt (20 ng) and 0, 10, 20 or 40 ng of 3Cwt. Cells were lysed for western blot analysis, as above

With the purpose of defining the precise cleavage sites of the FMDV proteases on *po*cGAS, co-transfection experiments were performed using a new construct with the FLAG-tag fused to the C-terminus of the protein (*po*cGAS-FLAG) and the resulting protein pattern was compared to that observed using 3xFLAG-*po*cGAS (with the N-terminal FLAG-tag). The results of co-transfecting HEK293 cells with 3Cwt, 3C(H46Y), Lbwt or Lb(C51A) with 3xFLAG-*po*cGAS or *po*cGAS-FLAG are shown in Fig. 2a and b, respectively. Expression of 3Cwt generated a “3xFLAG-cleavage product” of approximately 18 kDa (Fig. 2a) and a “cleavage product-FLAG” with an approximate molecular weight of 44 kDa (Fig. 2b). Comparative sequence analysis among 100 FMDV strains of the polyprotein junctions cleaved by 3C defined the P1-Q P1´-L/T/I and P1-E P1´-G/S as the residues typically recognized and cleaved by the protease [16]. Generation of the N-terminal 18 KDa and C-terminal 44 KDa fragments was consistent with cleavage taking place in the 130–170 stretch. When the swine cGAS sequence was analyzed in that region, positions Q140 and T141 were identified as meeting both 3C target sequence and cleavage products size criteria. Thus, we hypothesized that 3Cpro might be likely cleaving *po*cGAS between amino acids Q140 and T141. To confirm that, the threonine 141 was substituted by a proline in the *po*cGAS-FLAG plasmid. An equivalent substitution has been shown to impair VP3/VP1 cleavage by 3C on the FMDV polyprotein [36]. As shown in Fig. 2c, the introduction of the T141P mutation effectively abolished cleavage of *po*cGAS-FLAG by 3Cpro.

On the other hand, Lbpro co-expression generated a “3xFLAG-cleavage product” of about 40 kDa (Fig. 2a) and a “cleavage product-FLAG” of approximately 24 kDa (Fig. 2b). Though a consensus target sequence for Lpro has not been stablished, the recent identification of Lpro cleavage sites on several host proteins has revealed some sequence features shared among them like the enrichment in positively charged residues [14]. In the *po*cGAS sequence, a region matching those requirements and yielding N- and C-terminal fragments consistent with those observed, was identified, the K_322_VKNNLKRQ_330_ motif. Then, a mutagenesis analysis based on replacement of basic amino acids by negatively charged residues, proven effective in abolishing Lpro cleavage [37–39] was performed to address whether Lbpro was cleaving *po*cGAS at that site. Firstly, a mutant protein with substitutions K228E and R229E was generated, but we found that *po*cGAS K228E, R229E remained susceptible to cleavage by Lpro (Supplementary Fig. 2a). Then, a new mutation, Glutamine 330 to glutamic acid, was incorporated (*po*cGAS K228E, R229E, Q330E). When this last construct was tested we found that, although the cleavage efficiency by Lbpro seemed to be reduced, the triple mutant was still being degraded (Supplementary Fig. 2b). In order to further enrich the putative cleavage site region in negatively charged residues, two additional mutations (K322E and K324E) were added to generate *po*cGAS K228E, R229E, Q330E, K322E, K324E. When this mutant version of *po*cGAS (KVKNNLKRQ/EVENNLEEE) was co-expressed with Lbpro, we found it resistant to cleavage (Fig. 2d), proving that the K322-Q330 stretch was the Lpro target site on swine cGAS. A schematic representation of the FMDV proteases cleavage sites on the protein according to the tagged products obtained and mutagenesis analysis is shown in Fig. 2e. To further verify the proteases target sites and reproduce the context of infection in the presence of both proteases, the effect of increasing amounts of 3Cpro in HEK293 cells expressing Lbpro was analyzed. As shown in Fig. 2f, both 40 kDa and 18 kDa N-terminal 3xFLAG-*po*cGAS products could be simultaneously detected. The internal position of the 3C target site within the Lpro cleavage product could be also confirmed as increasing amounts of 3C progressively reduced the amount of the Lb-dependent 40 kDa product. Taken together, our results suggest that FMDV is redundantly degrading cGAS through the concomitant proteolytic activities of its two encoded proteases.

### Impact of cGAS degradation by the FMDV proteases on the antiviral response

**Fig. 3:**
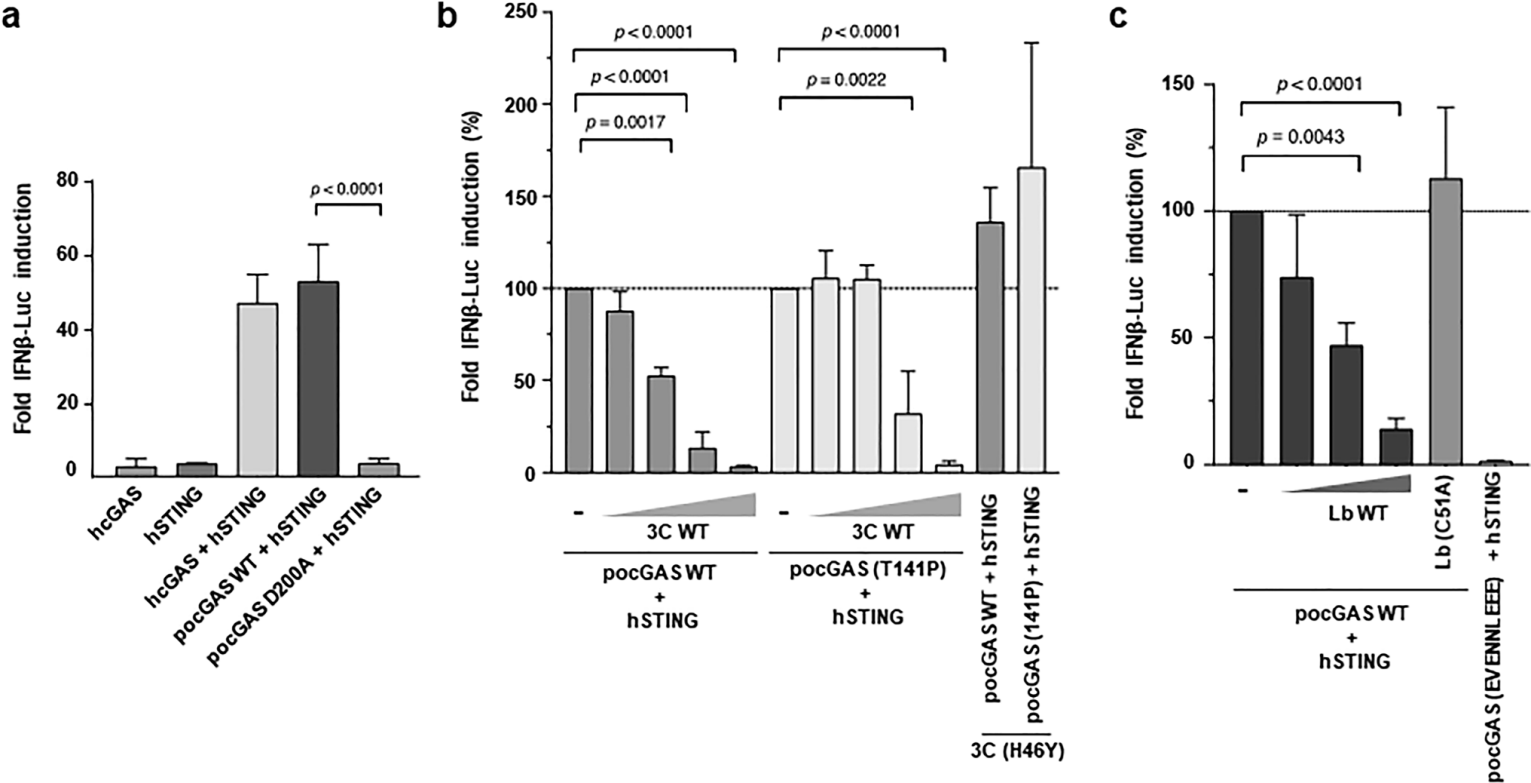
Impact of *po*cGAS cleavage by the FMDV proteases on IFN-β induction. **a**, **b**, **c** HEK293T cells (0.4 × 10^6^) were co-transfected with 25 ng of pIFN-β-FL, 12.5 ng of pRL-TK, 10 ng of plasmids expressing different versions of cGAS, *h*STING or both (**a**) and increasing amounts of 3Cwt (0.1, 0.5, 5 or 50 ng) (**b**) or Lbwt (0.1, 1 or 10 ng) (**c**). Inactive mutant proteases 3C(H46Y) (50 ng) and Lb(C51A) (10 ng) were also tested as controls. Cells were harvested 24 h after transfection for IFN-β assay. Data are mean ± SD of three independent assays. Significant differences (Student’s *t* test, *p* values) are indicated

To address the effect of cGAS cleavage by 3C and Lb proteases on the innate immune response against FMDV infection, we evaluated the result of their expression on the ability of the cGAS/STING pathway to induce the IFN-β promoter by an IFN-β reporter assay in HEK293T cells. For that, we first tested the stimulatory ability of the combination of *po*cGAS with the human STING (*h*STING) compared with that of the two components of human origin. As shown in Fig. 3a, *po*cGAS was fully active stimulating *h*STING in the assay when compared with the human cGAS. The specificity of the assay was further tested expressing a mutant *po*cGAS carrying substitutions reported to inactivate its cGAMP synthesis capacity, *po*cGAS (E200A/D202A) [40]. As expected, *po*cGAS (E200A/D202A) was unable to activate *h*STING for IFN-β induction (Fig. 3a). Next, we analysed how expression of increasing amounts of 3Cpro affected IFN-β induction when either *po*cGAS wt or the 3Cpro non-cleavable mutant T141P were co-expressed. As shown in Fig. 3b, 3Cpro reduced in a dose-dependent manner luciferase production promoted by *po*cGAS wt with a clear effect at the two lowest 3Cpro doses analysed (0.1 ng and 0.5 ng), while the *po*cGAS (T141P) mutant, which was fully active stimulating *h*STING, was clearly more resistant to the inhibitory effect of 3Cpro, requiring higher amounts of the protease (5 ng) to reduce luciferase expression. When 50 ng of 3Cpro were transfected (highest amount assayed), luciferase production was largely inhibited in both cases, most likely due to the expected inhibition of translation caused by 3Cpro rather than its proteolytic activity on *po*cGAS. At the same highest dose assayed (50 ng), expression of the inactive 3C(H46Y) mutant did not exert any inhibitory effect on *po*cGAS-stimulatory activity (Fig. 3b).

Following the same procedure, the effect of the FMDV Lbpro expression on the IFN-β induction through the cGAS/STING pathway was analysed (Fig. 3c). Co-expression of *po*cGAS wt with increasing amounts of Lbpro induced a dose-dependent reduction in IFN-β transcription. As above, the highest dose assayed of the catalytically inactive form Lb(C51A) did not affect the *po*cGAS-stimulatory activity. In this case, the effect of Lbpro on the *po*cGAS mutant resistant to cleavage by Lpro (KVKNNLKRQ/EVENNLEEE) could not be tested as this construct was deficient in STING stimulation for IFN-β induction (Fig. 3c). Interestingly, the amino acid equivalent to K322 in the human and mouse sequence is conserved (*h*K347 and *m*K335, respectively). K347 contributes to the B DNA-binding site of human cGAS and SUMOylation of *h*K347 and *m*K335 is known to impair cGAS ability to bind to DNA and hinder its dimerization [5, 41]. Given the functional relevance of the human and mouse counterparts of K322 it appears likely that the substitutions made in that stretch to eliminate the Lpro cleavage site impacted negatively on *po*cGAS functionality. Altogether, these results confirm the ability of the FMDV-encoded 3C and Leader proteases to efficiently counteract cGAS/STING-mediated type I IFN production.

### Inhibitors of the cGAS/STING pathway promote FMDV infection

**Fig. 4:**
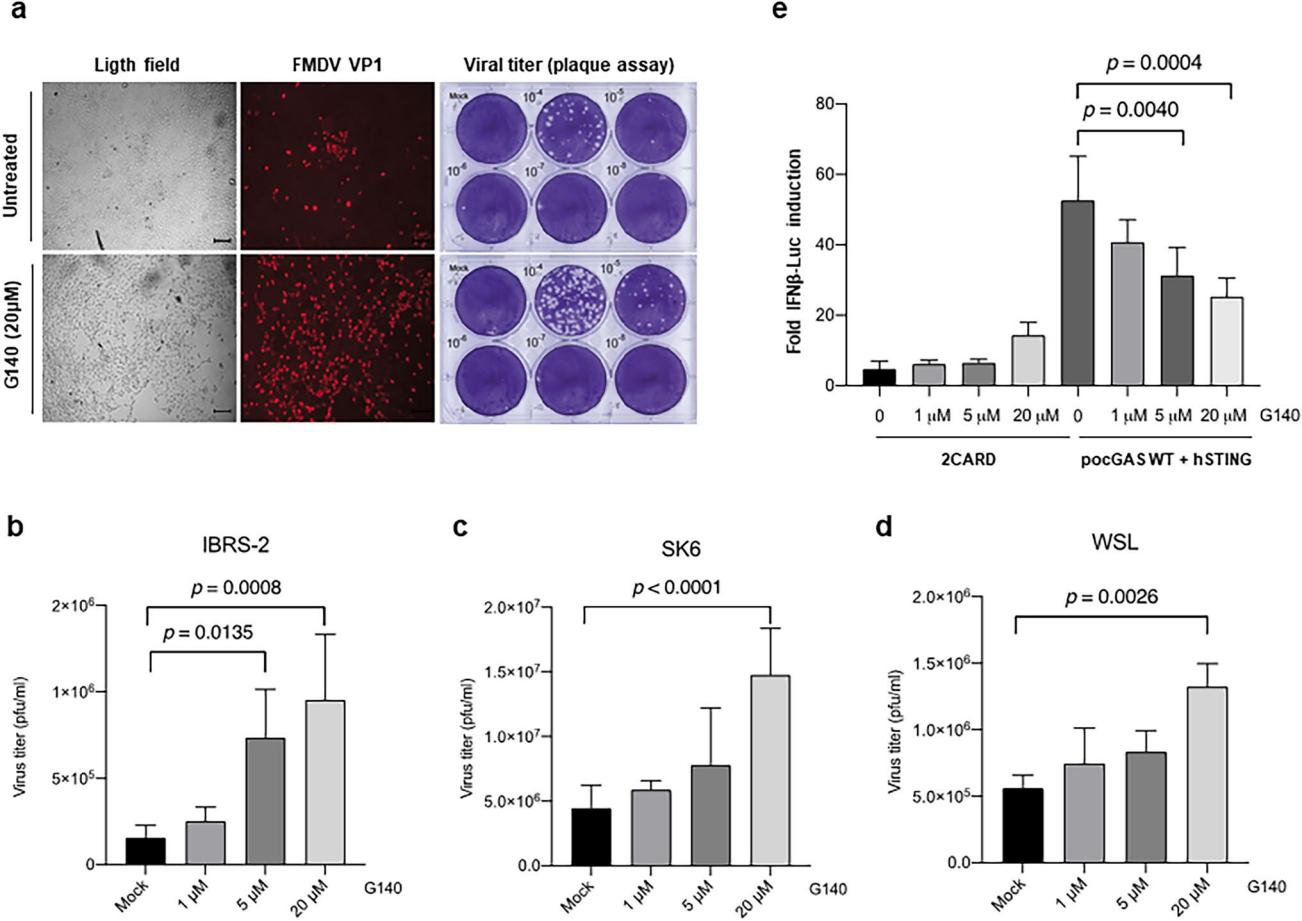
Treatment with the cGAS inhibitor G140 promotes FMDV infection. **a** IBRS-2 cells (2 × 10^5^) were infected with FMDV O1K at an MOI of 0.01. One hour later, infection medium was replaced with fresh medium containing 20 µM G140 and cells were further incubated for 18 h. Then, supernatants were collected and cells were fixed for VP1 expression analysis (red) in comparison with non-treated cells under a wide field fluorescence microscope (left panel); scale bars, show 100 µm. Viral titers in the supernatants (tenfold dilutions) were quantified by plaque assay (right panel). **b**, **c**, **d** IBRS-2 (**b**), SK6 (**c**) or WSL (**d**) cells were infected and treated with increasing concentrations of G140 as in (**a**) and 18 h later the supernatants were collected and viral titers were determined by plaque assay. Data are mean ± SD of at least three independent assays performed in duplicate. **e** HEK293T cells (0.4 × 10^6^) were co-transfected with 25 ng of pIFN-β-FL, 12.5 ng of pRL-TK and either 2 ng of 2CARD or *po*cGAS/*h*STING (10 ng each). After 7 h, G140 was added to the medium at the indicated concentrations and cells were incubated for 24 h. Then, cells were harvested for IFN-β assay. Data are mean ± SD of three independent assays. Significant differences (one-way ANOVA, *p* values) are indicated

Having seen that cGAS is a target for the FMDV-encoded proteases and the counteracting effect of 3C and Lpro cleavage on the antiviral response induced by the cGAS/STING pathway, we first studied the effect of treatment with the cGAS inhibitor G140 on FMDV infection in swine cells. G140 has been reported as a potent inhibitor of human and murine cGAS by direct binding to the cGAS active site and inhibiting its enzymatic activity competitively [42, 43]. For that, swine IBRS-2 cells were infected at an MOI of 0.01 for 18 h in the presence or absence of 20 µM G140 in the culture medium and then cell monolayers were analysed by fluorescence microscopy using an anti-FMDV VP1 antibody and supernatants were collected for determination of viral titers. As shown in Fig. 4a, a higher accumulation of VP1 and an increase of about 1 log in viral titers was observed as a result of treatment with G140, compared with non-treated cells (Fig. 4a, lower and upper panels, respectively). To further confirm the enhancing effect of G140 on FMDV infection, increasing amounts of the drug were added to the culture medium and FMDV infection led to proceed as above in three different swine cell lines: IBRS-2, SK6 and WSL. The resultant viruses in the supernatants were quantified by plaque assay 18 h later and are shown in Fig. 4b, c and d, respectively. A G140 dose-dependent enhancing effect on FMDV titers was observed in all three cell lines, with significant differences at 20 µM G140 (and also at 5 µM for IBRS-2 cells). Consistently, G140 exerted a dose-dependent inhibitory effect on the induction of IFN-β via cGAS/STING in the reporter assay, while induction via RLR exerted by a constitutively active RIG-I mutant (2CARD) remained unaffected by treatment with G140 (Fig. 4e). The lack of cell toxicity of the different concentrations of G140 tested was confirmed by MTT assay in the three swine cell lines and HEK293T cells (Supplementary Fig. 3a, b, c, d). Together, these data indicate that chemical inhibition of cGAS with G140 favours FMDV infections in porcine cells.

**Fig. 5:**
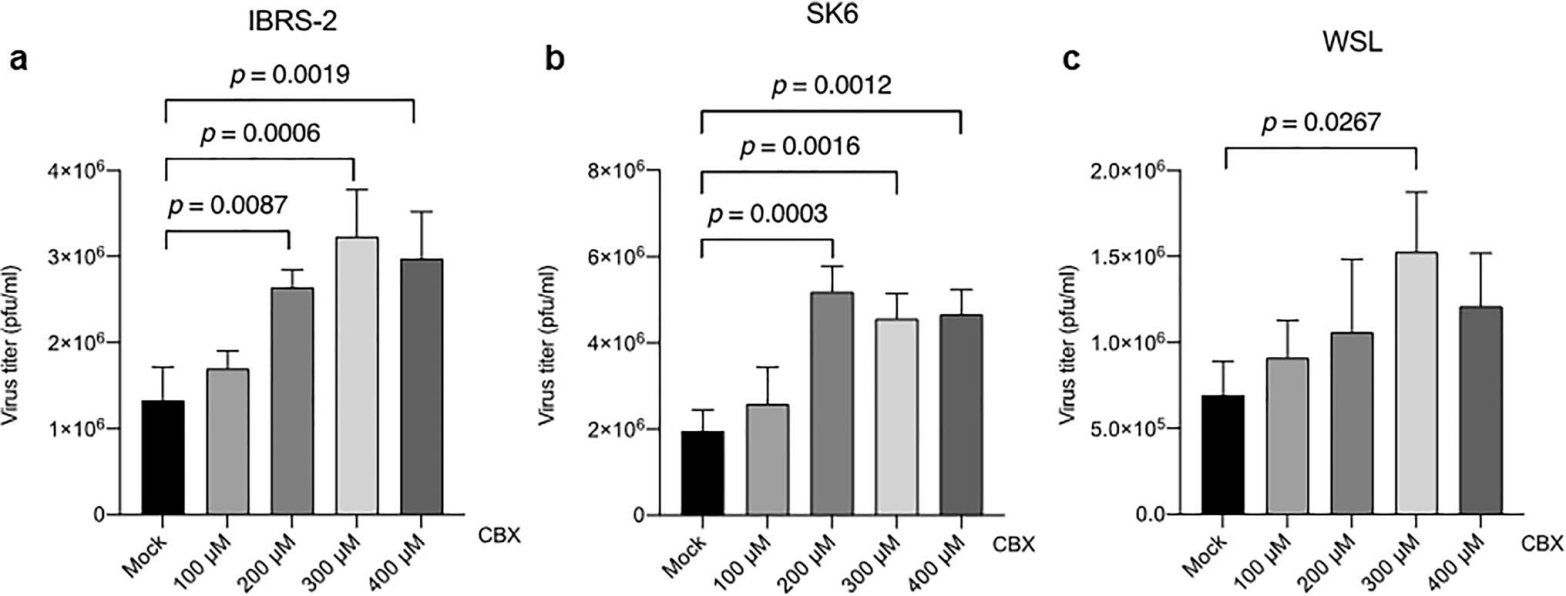
FMDV infection is enhanced by treatment with the gap-junction inhibitor carbenoxolone (CBX). IBRS-2 (**a**), SK6 (**b**) or WSL (**c**) cells (2 × 10^5^) were treated with CBX at different concentrations for 3 h and then infected with FMDV O1K at an MOI of 0.01. Supernatants were collected 18 h after infection and viral titers were determined by plaque assay. Data are mean ± SD of three independent assays performed in duplicate. Significant differences (one-way ANOVA, *p* values) are indicated

Experimental evidence suggests that cGAMP can be transmitted across gap junctions from producer cells to adjacent cells, contributing to accelerated immune responses [44, 45]. With the purpose of evaluating whether carbenoxolone (CBX), a connexin inhibitor that blocks gap junctions preventing cell-to-cell transfer of cGAMP in vitro [44, 46, 47], could interfere with FMDV infection, we treated IBRS-2, SK6 and WSL cells with increasing amounts of CBX for 3 h before infection with FMDV at an MOI of 0.01 for 18 h. As shown in Fig. 5a, b, c, CBX treatment promoted FMDV infection in all three cell lines, with maximum effects at 200–300 µM. Though requiring further research, these results point in the same direction as those obtained with dengue or influenza viruses [28, 46] suggesting that the blockade of gap junctions may prevent the intercellular transmission of cGAMP and consequently favouring viral spread.

As a whole, the enhancing effect on FMDV infection observed for the two different compounds tested, known to interfere with the cGAS/STING pathway at different steps, highlights the relevance of this DNA-sensing antiviral route against FMDV infection. This is in agreement with a recent work showing that over-expression of cGAS in porcine kidney cells (PK15) reduced FMDV replication [31]. We further confirmed the key role of the cGAS enzymatic activity for restraining FMDV infection using recombinant *po*cGAS-eGFP proteins that were overexpressed either in the wild-type version or as the E200A/D202A inactive form in swine IBRS2 and WSL cells that were subsequently infected with FMDV at different MOI (Supplementary Fig. 4a, b). Our results clearly show a delay in infection in cells expressing the active form of *po*cGAS compared to its inactive counterpart.

### Detection of endogenous cGAS cleavage in WSL cells

**Fig. 6:**
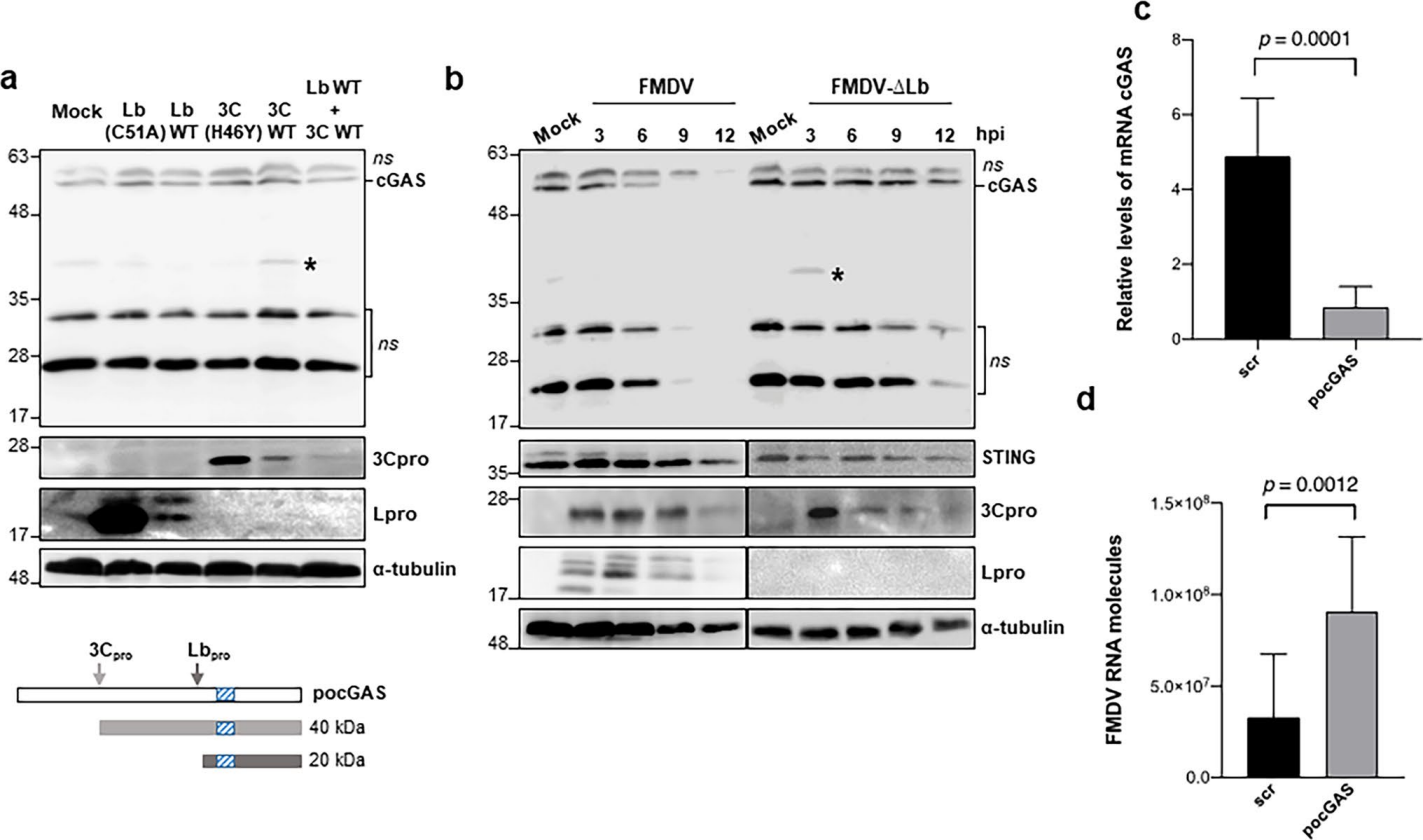
Endogenous *po*cGAS is cleaved by the FMDV proteases and *po*cGAS knockdown enhances FMDV infection in WSL cells. **a** WSL cells (2 × 10^5^) were mock-transfected or transfected with 100 ng of Lb(C51A), Lbwt, 3C(H46Y), 3Cwt, or Lbwt together with 3Cwt (50 ng each). Cells were lysed 24 h after transfection and analysed by immunoblot for the indicated proteins. The approximate expected size of the cleavage products enclosing the region recognised by the anti-cGAS antibodies (blue box) are indicated below. **b** WSL cells were mock-infected or infected with FMDV O1K (MOI of 5) or FMDV-ΔLb (MOI of 10) and collected at different times after infection for western blot analysis for the indicated proteins. The cleavage products generated by 3C are indicated with an asterisk; ns, non-specific bands. **c** WSL cells were transfected with 200 nM of a specific *po*cGAS siRNA or a scramble siRNA (scr) for 48 h. Then, cells were collected and the relative levels of *po*cGAS mRNA quantified by RT-qPCR. **d** WSL cells were transfected with siRNAs for 48 h as in (**c**) and then infected with FMDV O1K at an MOI of 0.01 for 8 h. The FMDV RNA molecules in siRNA-transfected/infected cells were quantified by RT-qPCR. Data are mean ± SD of two independent transfection assays and sextuplicate quantification of each cDNA. Significant differences (Student’s *t* test, *p* values) are indicated

Having precisely mapped the cleavage sites of the 3C and Lb proteases, we tried detection of swine cGAS cleavage products generated during FMDV infection in WSL cells, derived from wild boar lung cells with a macrophage linage origin with the loss of some specific myeloid markers [48]. As shown in Figs. 1a, 6a, b, in WSL cells *po*cGAS could be detected as a neat band allowing to assay the effect of the proteases, as well as infection on the endogenously expressed *po*cGAS. When the catalytically active 3Cpro was expressed in WSL cells, a band of approximately 40 kDa was observed, consistent with the predicted size for the C-terminal *po*cGAS fragment after cleavage by 3C at Q141/T142 (Fig. 6a). However, the fragment of about 20 kDa expected to be generated by Lbpro cleavage could not be detected under our experimental conditions. Interestingly, when both proteases were co-expressed, the cleavage product of 3Cpro, which can also be cleaved by Lbpro, was not detected (Fig. 6a). Next, WSL cells were infected with either FMDV derived from a full-length cDNA clone or with a mutant FMDV generated from the same cDNA clone in which the Lb-coding sequence was deleted (FMDV-∆Lb). As illustrated in Fig. 6b, infection of WSL cells with FMDV at an MOI of 5 resulted in *po*cGAS degradation, from 6 h post-infection (pi) onwards, and being complete at 9 h pi. In contrast, in cells infected with FMDV-∆Lb at an MOI of 10, *po*cGAS could be clearly detected at 12 h pi. Notably, at 3 h pi with an FMDV with a disabled Lpro, a band of migration corresponding to the C-terminal *po*cGAS product after 3C cleavage (~ 40 kDa) could be detected (Fig. 6b). Equivalent results were obtained in WSL cells treated with inhibitors of the caspase, proteasomal or lysosomal pathways after 3 h of infection with FMDV or FMDV-∆Lb (Supplementary Fig. 5a, b). It is worth mentioning that Yan et al. [30] also failed to detect the swine cGAS C-terminal cleavage fragment generated by 3Cpro during SVV infection in HEK293T cells stably expressing cGAS/STING. In our case, the combined cleavage by 3C and Lpro may contribute to the difficulty in detecting the cGAS fragments derived from the activity of the FMDV-encoded proteases.

Finally, to analyse the effect of cGAS deficiency on FMDV infection in WSL cells, its endogenous expression was silenced by RNAi. For that, a cGAS-specific siRNA or a scrambled control siRNA were transfected into WSL cells that were infected 48 h later with FMDV at an MOI of 0.01 for 8 h. The levels of cGAS mRNA and the FMDV RNA molecules were quantified by RTqPCR (Fig. 6c, d, respectively). Knockdown of cGAS in WSL (Fig. 6c) enhanced FMDV replication and a 17.5-fold increase in viral RNA could be measured at 8 h pi in cells treated with *po*cGAS-specific siRNAs (Fig. 6d).

Together, our results highlight the relevance of cGAS as a restriction factor for FMDV infection in swine cells, and show that FMDV has developed a counteracting strategy based on the double targeting of the DNA sensor for direct cleavage by the virally encoded 3C and Leader proteases.

## Discussion

As a consequence of viral infection, the host antiviral defences are rapidly activated to prevent viral replication and spread. FMDV replicates its RNA genome in the cytosol where innate immune RNA sensors recognize foreign RNA molecules derived from infection to activate the RLR/MAVS or TLR routes and induce the innate immune response in the host [11, 49]. Mitochondrial DNA released to the cytoplasm as a consequence of FMDV infection has also been identified as an activator of innate immunity trough the cGAS/STING route [31]. The FMDV 3C and L proteases have a pivotal role in counteracting the host antiviral response by targeting proteins involved in sensing viral infection and triggering or amplifying the innate immune response [11, 14, 49, 50].

In this work, we describe the double cleavage of swine cGAS by the two FMDV-encoded proteases and provide evidence suggesting endogenous cGAS degradation at early times during FMDV infection. A recent report suggested that FMDV infection leads to the degradation of STING mediated by nonstructural protein 2B through a non-characterized mechanism, while not affecting cGAS integrity in PK15 cells [31]. We did not observe an evident STING degradation under our experimental conditions other than a decay at late times after infection (around 9–12 h pi) when translation is severely compromised [51]. However, taking into account that autophagy triggered by RE stress during FMDV infection has been associated with STING degradation [52], the contribution of this pathway to the decay of STING observed at late times after infection cannot be ruled out. Though detection of endogenous swine cGAS is known to be difficult [30], a rapid decrease in the protein levels at early times after infection (3–6 h pi) could be detected in SK6 and WSL cells. When WSL cells were infected with an Lpro-deficient FMDV mutant, the stability of the protein was significantly increased, confirming the role of the Leader protease in cGAS degradation. A similar approach for 3C is not feasible due to the essential role of 3C in processing the polyprotein and being the deletion of 3C lethal for the virus. However, a product consistent with the cGAS 3C cleavage product could be detected during infection with the Lpro-deficient FMDV, suggesting the 3C cleavage of cGAS during infection. Additional research will be needed to address whether the enzymatic activity of one protease may become predominant over the other at specific stages of infection and to assess the relevance of the cGAS levels and accessibility in the cytoplasm of infected cells over time. Unfortunately, those studies are currently limited by the few available reagents with cross-reactivity with swine cGAS.

By co-expression assays, we were able to precisely identify the cleavage sites for each protease. Though the autophagy-dependent degradation of swine cGAS during SVV infection had been suggested [31], while this work was in preparation, the cleavage of cGAS by the 3C protease of SVV between K138 and L139 was reported [30]. Interestingly, we identified the FMDV 3Cpro cleavage site on swine cGAS between Q140 and T141, partly overlapping with the specific motif W_137_KLQ_140_ defined as SVV 3C target site, and consistent with the specificity differences documented for the 3C proteases encoded by different picornaviruses [53]. Moreover, these results suggest that different swine picornaviruses may have evolved converging strategies for counteracting the host innate response triggered by the cGAS/STING pathway. In the case of Lpro, the motif KVKNNLKRQ comprising residues 322–330 in swine cGAS was identified as the cleavage site. This target sequence displays some similarity with the Lpro self-cleavage site at the L/P1 junction in the FMDV polyprotein and also, but to a lesser extent, with other Lpro target sequences identified to date [14]. As there is no consensus sequence for Lpro cleavage site, our results expand the list of reported Lpro target sequences, helping in the identification of new host cell proteins as putative Lpro targets. By analogy to the better characterized human cGAS, cleavage by 3Cpro would excise the N-terminal disordered region of cGAS (1–134 in *po*cGAS and 1–160 in *h*cGAS) known to bind DNA and enhance cGAMP production [54]. Although the C-terminal nucleotidyltransferase domain (NTase) is sufficient to synthesize cGAMP in the presence of dsDNA [55], Bhattacharya et al. showed using an in vitro recombination system that truncation of the N-terminal domain reduced the affinity and competed for binding of the C-terminal NTase domain to dsDNA inhibiting cGAMP production [29]. Cleavage by Lpro at positions 322–330 would release the C-terminal zinc ribbon/thumb domain and several residues involved in two DNA-binding sites (termed A and B sites), all required for activity [5] (Fig. 2e).

The impact of cGAS cleavage by either protease dampening the IFN-I response was demonstrated, stressing the importance of counteracting the cGAS-dependent DNA sensing signaling route for the FMDV viral cycle. We were able to circumvent the inhibitory effect exerted by the expression of *po*cGAS on FMDV replication when a mutant protein (E200A/D202A) defective for cGAMP synthesis was overexpressed instead. To further verify the physiological relevance of the cGAS/STING signaling axis for FMDV infection, the effect of specific inhibitors of this route was tested in three different swine cell lines susceptible to FMDV. Treatment with either the cGAS inhibitor G140 or with a drug known to block intercellular transmission of cGAMP through gap-junctions (CBX) significantly favored the progress of FMDV infection in all three cell lines assayed. Analysis of the cGAMP levels during FMDV infection might be useful to gain insight on the physiological effect of cGAS specific cleavage and will be addressed in future work. The relevance of cGAMP in stimulation of innate immunity beyond the producer cells is progressively increasing [56] and packaging of cGAMP in viral particles and extracellular vesicles has been documented [57]. Also, poxviruses are known to produce “poxins” that degrade cGAMP for immune evasion [23]. Moreover, restriction of RNA viral infection by cGAS via a non-canonical pathway independent of cGAMP production and leading to IFN-β transcription has been recently described, supporting the pleiotropic inhibitory effect of cGAS on the replication of RNA viruses [58]. Our results provide new information on the functional implications of an active cGAS/STING pathway against FMDV infection in swine cells and illustrates how FMDV in turn is actively counteracting cGAS antiviral activity by redundantly targeting the protein for cleavage by the virally encoded Leader and 3C proteases. A better understanding of how RNA viruses interact with and subvert the cGAS/STING pathway will be essential for developing improved antiviral therapies.

## Materials and methods

### Cell lines and transfection

Human kidney cell lines HEK293 and HEK293T (both from ATCC), BHK-21 cells (ATCC), swine kidney cell lines SK6 and IBRS-2 (from Centro de Investigación en Sanidad Animal, INIA-CSIC, Spain) and wild boar lung cell line WSL [59] (FLI, Germany) were cultured in Dulbecco’s modified Eagle’s medium (DMEM) (Gibco, Thermo Fisher Scientific) supplemented with 5% fetal bovine serum (FBS) at 37 °C with 5% CO_2_. Subconfluent monolayers of the different cell lines were transfected with plasmids using Lipofectamine 2000 (Invitrogen, Thermo Fisher Scientific) and Opti-MEM I reduced serum medium (Gibco, Thermo Fisher Scientific) following the manufacturer’s instructions. In some experiments, the transfection medium was supplemented with 20 μM zVAD-FMK (Promega), 10 μM MG132 (Cayman Chemical) or 50 µM chloroquine (CQ, Sigma-Aldrich) or 20 μM puromycin (Sigma-Aldrich).

### Plasmids

Plasmid expressing recombinant 3xFlag-porcine cGAS (3xFlag-*po*cGAS) [40] was a kind gift from Bei-Bei Chu. Plasmids encoding FMDV Lbwt and Lb(C51A) have been previously reported [38]. FMDV 3Cwt was generated by PCR amplification of the corresponding region from an FMDV O1K full-length cDNA clone [60] and insertion into the BamHI and XbaI sites of pcDNA3.1(+) (Invitrogen, Thermo Fisher Scientific). FMDV 3C(H46Y) was generated by site-directed mutagenesis on the FMDV 3Cwt construct (NZYMutagenesis, nzytech). FMDV-ΔLb was generated from the FMDV O1K full-length cDNA clone in which the Lbpro sequence was deleted. For that, the 1369-bp KpnI fragment spanning positions 581–1950 was replaced by a KpnI synthetic DNA fragment carrying deletion of nt 801–1316 (encoding for Lbpro). Sequence numbers correspond to FMDV O1K GenBank accession number X00871.1. Plasmids expressing human cGAS and human STING were kindly provided by Ana Fernández-Sesma. Porcine-cGAS-Flag (*po*cGAS-Flag) was made by insertion of the porcine cGAS sequence amplified by PCR from 3xFlag-*po*cGAS into the Hind III/Mlu I sites of pCMV6-Entry plasmid (Origene). Constructs expressing cGAS mutants E200A/D202A, T141P, or KVKNNLRKQ to KVKNNLEEQ, KVKNNLEEE, or EVENNLEEE, respectively were made by site-directed mutagenesis on *po*cGAS-Flag (NZYMutagenesis, nzytech). Plasmids expressing *po*cGAS-eGFP or *po*cGAS (E200A/D202A)-eGFP were generated by replacing their Flag sequences in pCMV6-pcGAS-Flag or pCMV6-pcGAS(E200A/D202A)-Flag by that of eGFP using the Mlu I and Sac II sites.

Primers used in generating the above plasmids used in this study are listed below:

F_ Hind III-pcGAS: 5′- GGCGAAGCTTACCATGGCGGCCCGGCGG-3′.

R_ Mlu I-pcGAS: 5′-CTCGACGCGTCCAAAAAACTGGAAATCC-3′.

F_ pcGAS T141P: 5′-CCTGGAAGCTCCAGCCGGTGCTGGAGAAG-3′.

R_pcGAS T141P: 5′-CTTCTCCAGCACCGGCTGGAGCTTCCAGG-3′.

F_pcGAS (KR-EE): 5′-GGAGCAAAAGTTAAAAACAATCTGGAAGAACAGCCA.

TTTTACCTGGTACCCAA-3′.

R_pcGAS (KR-EE): 5′-TTGGGTACCAGGTAAAATGGCTGTTCTTCCAGA.

TTGTTTTTAACTTTTGCTCC-3′.

F_pcGAS (KRQ-EEE): 5′-GCAAAAGTTAAAAACAATCTGGAAGAAGAGCCATT.

TTACCTGGT-3′.

R_pcGAS (KRQ-EEE): 5′-ACCAGGTAAAATGGCTCTTCTTCCAGATTGTTTT TAACTTTTGC-3′.

F_pcGAS (KVK-EVE): 5′-CAGTCAGTGGCTTGGAGCAGAAGTTGAAAACAAT CTGGAAGAAGA-3′.

R_pcGAS (KVK-EVE): 5′-TCTTCTTCCAGATTGTTTTCAACTTCTGCTCCAAG CCACTGACTG-3′.

F_pcGAS (E200A/D202A): 5′-GTGAAGATTTCTGCTCCCAATGCATTTGCTGT TATGTTCAAACTGGAAGT-3′.

R_pcGAS (E200A/D202A): 5′-ACTTCCAGTTTGAACATAACAGCAAATGCATT GGGAGCAGAAATCTTCAC-3′.

F_3C: 5′-AGCTAGGATCCTGATTGTCATGGAGAGTGGTGC-3′.

R_3C: 5′-ACAATCTAGACCTCGTGGTGTGGTTCGGGGTCAATG-3′.

F_3CH46Y: 5′-GCTTACCTCGTGCCTCGTTATCTCTTCGCAGAGAAG-3′.

R_3CH46Y: 5′-CTTCTCTGCGAAGAGATAACGAGGCACGAGGTAAGC-3′.

F_pcGAS-Mlu I-eGFP: 5′-TTTTGGACGCGTATGGTGAGCAAGGGCG-3′.

R_Sac II-eGFP: 5′-CTATGACCGCGGTTACTTGTACAGCTCGTC-3′.

### Viruses and infection

Viral titers were determined by plaque assay and expressed as plaque-forming units (pfu)/ml. FMDV O1BFS isolate was propagated in SK6 cells and the titer of the viral stock was 3 × 10^7^. FMDV O1K and FMDV-ΔLb derive from a full-length cDNA clone [60] and were propagated in BHK-21 cells and the titers of viral stocks were 1 × 10^8^ pfu/ml and 2 × 10^7^ pfu/ml, respectively. Sequencing analysis confirmed the presence of the Lb deletion in the FMDV-ΔLb viral stock. The effect of treatment with the cGAS inhibitor G140 (inh-g140, InvivoGen) on FMDV infection was performed by adding different amounts of the compound in the culture medium of cells infected with 0.01 pfu/cell 1 h after infection during 18 h. Then, the supernatants were collected for determination of viral titers by plaque assay. The assay with the inhibitor of gap-junctions CBX (C4790, Sigma-Aldrich) was made by adding different amounts of the compound to the culture medium for 3 h. Then, the medium was removed and cells were infected with 0.01 pfu/cell of FMDV. At 18 h pi, the supernatants were collected for quantification of viral titers.

### Western blotting

Cells were collected in sample buffer (62.5 mM Tris–HCl, pH 6.8, 2% SDS, 0.1 M DTT, 17% glycerol, and 0.024% bromophenol blue) and boiled for 5 min. The proteins were separated by electrophoresis on SDS–polyacrylamide gels, transferred to nitrocellulose membranes and detected by specific antibodies. Primary antibodies used were: anti-cGAS (PA5-43,855, Invitrogen), anti-STING (19,851-1-AP, Proteintech), anti-Flag (F7425, Sigma-Aldrich), anti-α-Tubulin (T5168, Sigma-Aldrich), anti-eIF4GI [61], anti-FMDV Lpro [62]; anti-FMDV 3C (2D2) and anti-FMDV type-O VP1 (B2) antibodies were a gift from Emiliana Brocchi. Horseradish peroxidase-conjugated goat anti-rabbit, or goat anti-mouse (Thermo Fisher Scientific) IgG were used for detection of membrane-bound proteins. Protein bands were visualized with the ECL detection system (Perkin Elmer Plus-ECL) by exposure to X-ray films or digitalized using the ImageQuant LAS 4000 system (GE Healthcare). The original blots corresponding to the results presented in this work are shown in Supplementary Fig. 6.

### IFN-β reporter assay

HEK293T cells (0.4 × 10^6^) were co-transfected with 25 ng of a reporter plasmid expressing firefly luciferase under the *h*IFN-β promoter (pIFN-β-FL, a gift from A. García-Sastre), 12.5 ng of renilla luciferase reporter plasmid (pRL-TK) (Promega), 10 ng of plasmids expressing different versions of cGAS, *h*STING or both. The effect of the FMDV proteases on the cGAS/STING-dependent IFN-β induction was tested co-transfecting plasmids expressing Lbwt (0.1, 1 or 10 ng), Lb(C51A) (10 ng), 3Cwt (0.1, 0.5, 5 or 50 ng) or 3C(H46Y) (50 ng). Cells were harvested 24 h after transfection and IFN-β induction measured (Dual-Luciferase Reporter Assay, Promega). To test the effect of treatment with the cGAS inhibitor G140, cells were transfected as above with *po*cGAS and *h*STING (10 ng each) or with 2 ng of the RIG-I constitutively active 2CARD mutant (provided by A. García-Sastre). After 7 h, G140 was added to the medium at 1, 5 or 20 µM and cells were incubated for 24 h. Then, cells were harvested for IFN-β assay, as above. Firefly luciferase activity was normalized to renilla and expressed as fold differences relative to mock-transfected cells.

### Immunofluorescence

IBRS-2 or WSL cells were cultured in 24-well plates and transfected with plasmids expressing *po*cGAS-eGFP or *po*cGAS (E200A/D202A)-eGFP (800 ng per well) for 24 h. Then, cultures were infected with FMDV O1K at different MOI. At 7, 18 or 24 h after infection, cells were fixed with 4% paraformaldehyde for 15 min at RT, permeabilized with 0.02% Triton X-100 in 1X PBS for 10 min and stained with anti-VP1 and anti-mouse Alexa-555 as primary and secondary antibodies, respectively. Then, cells were directly analysed under a wide field fluorescence microscope (Leica Application Suite V4.12). In some experiments, after 1 h of infection, the medium was replaced by fresh medium containing 20 µM G140 and cells further incubated for 18 h and then fixed, stained and observed as above.

### Cell viability assay

The cytotoxic effect of G140 or CBX treatment in the different cell lines used in this study was analyzed by [4,5-dimethyl-2-thiazolyl]-2,5-diphenyl-2-H-tetrazolium bromide (MTT) assay. For that, cells were seeded on 96-well plates and incubated for 24 h before treatment. Then, the culture medium was replaced with fresh medium containing 1, 5 or 20 µM G140 (for HEK293T, IBRS-2, SK6 and WSL cells) or 100, 200, 300 or 400 µM CBX (for IBRS-2, SK6 and WSL cells). Cells treated with G140 were incubated for 24 h and those treated with CBX for 3 h at 37 °C. Then, 90 μl of each supernatant was removed and replaced with fresh medium containing 10 µl of 7.5 mg/ml MTT in PBS. Cells were further incubated at 37 °C in the dark for 2 h and lysed at RT in lysis buffer (20% SDS, 0.45N acetic acid and 0.025 N HCl in N, N-dimethylformamide) for 15 min. The absorbance at 550 nm was recorded using a microplate reader (Bio-Rad). Data are mean ± standard deviation of six independent assays.

### siRNA-induced cGAS silencing

Chemically synthesized 21-nt siRNA duplexes targeting swine cGAS (5-UGAAAUUGAUGGAGUGAAAUU-3′) or swine scramble siRNAs [63] were purchased from Dharmacon. WSL cells were seeded in 12-well plates and transfected for 48 h with 200 nM siRNAs using Lipofectamine 2000. Then, cells were infected with 0.01 pfu/cell of FMDV O1K for 8 h. Total RNA was extracted and the expression levels of *po*cGAS and the FMDV RNA molecules were quantified by RT-qPCR.

### RNA extraction and RT-qPCR

Total cytoplasmic RNA was extracted using Tri-Reagent (Sigma) and DNase-treated with Turbo DNA-free kit (Ambion) according to the manufacturer’s recommendation. The RNA was quantified by spectrophotometry using a NanoDrop (ND-1000, Thermo Fisher) and 500 ng of RNA was used for RT with SuperScript III Reverse Transcriptase (Invitrogen) at 55 °C for 30 min. RT aliquots (1/10) were used for qPCR using LightCycler FastStart DNA Master SYBR green I (Roche) [64]. For amplification of porcine cGAS mRNA the following primers were used: KD pocGAS-F: 5′-TGGAGGCTTTCCTTCTCTCA-3′ and KD pocGAS-R: 5′-TGAGGGTCCTGGGTACAGAC-3′. Relative expression of cGAS was calculated using the ΔΔCT method normalizing to GAPDH. The FMDV RNA molecules per ng of cytoplasmic RNA was determined as previously described [64] by standard curves generated with RNA transcribed from the FMDV O1K cDNA clone.

### Statistical analysis

The statistical analysis was performed using one-way analysis of variance (ANOVA) or the unpaired two-tailed Student’s *t* test. *p* values were calculated using Graphpad Prism v9. Data were expressed as mean ± SD.

### Supplementary Information

Below is the link to the electronic supplementary material.

Supplementary file1 (TIF 118 KB)

Supplementary file2 (TIF 167 KB)

Supplementary file3 (TIF 82 KB)

Supplementary file4 (TIF 528 KB)

Supplementary file5 (TIF 197 KB)

Supplementary file6 (PDF 823 KB)

## Data Availability

The data and materials will be available from the corresponding author upon reasonable request.
