# Supplementary figures and images for: The antiviral response triggered by the cGAS/STING pathway is subverted by the foot-and-mouth disease virus proteases
Source: Cell Mol Life Sci. 2024 Mar 20;81(1):148. doi: 10.1007/s00018-024-05190-7 (PMC10954996; doi:10.1007/s00018-024-05190-7)

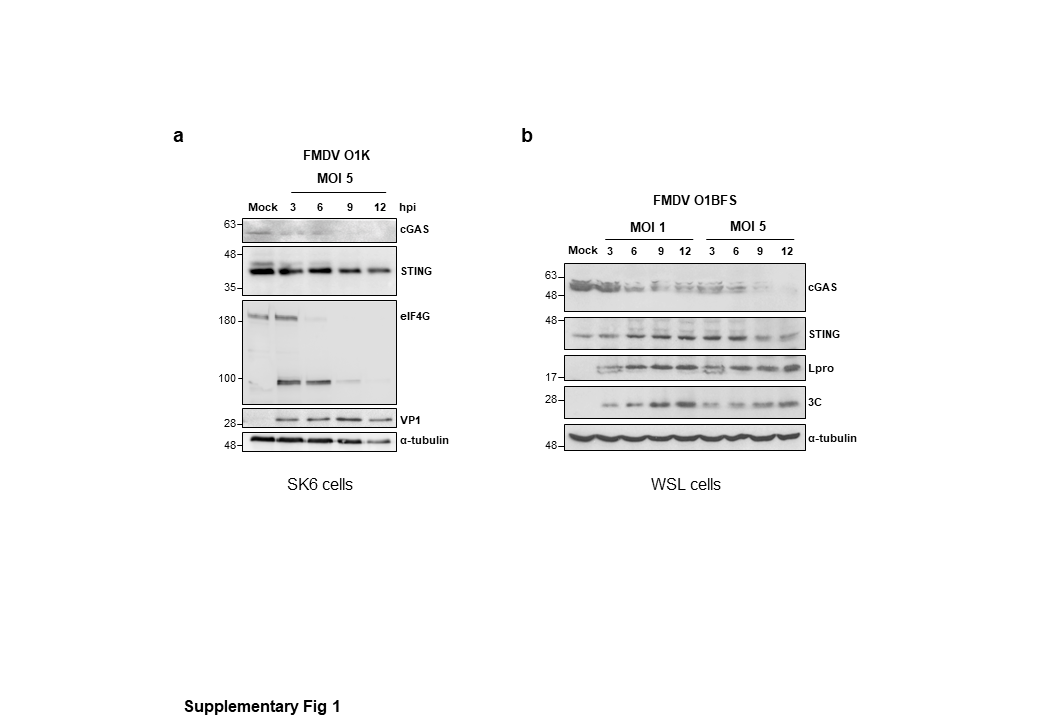

Supplement: Supplementary file 1 — Supplementary file1 (TIF 118 KB) [file 18_2024_5190_MOESM1_ESM.tif]

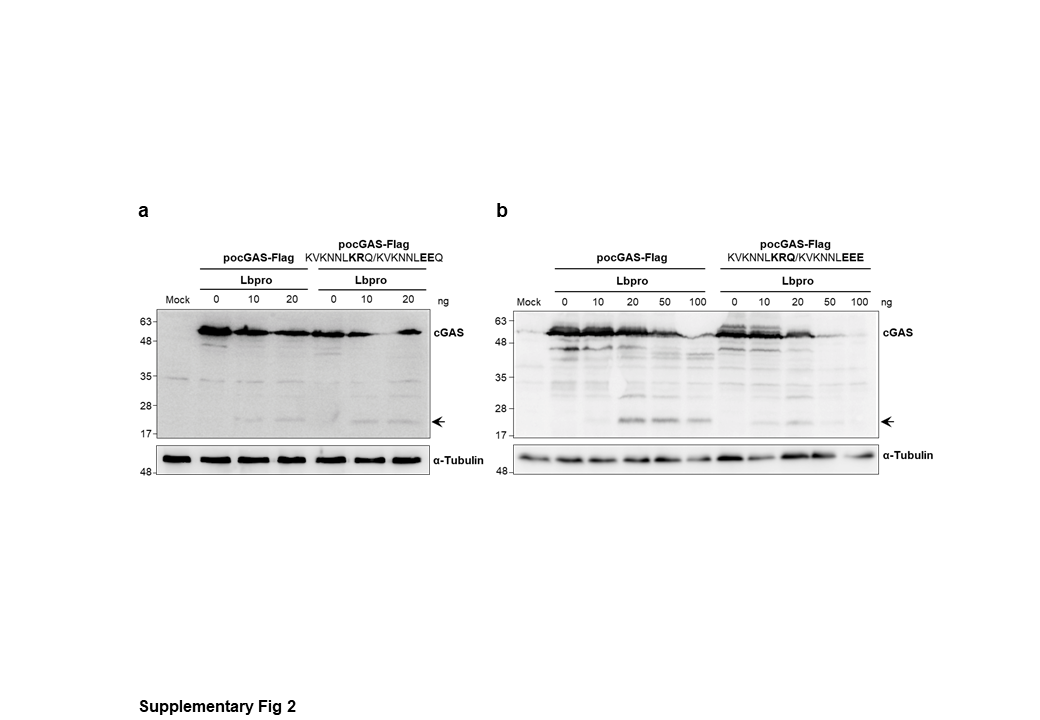

Supplement: Supplementary file 2 — Supplementary file2 (TIF 167 KB) [file 18_2024_5190_MOESM2_ESM.tif]

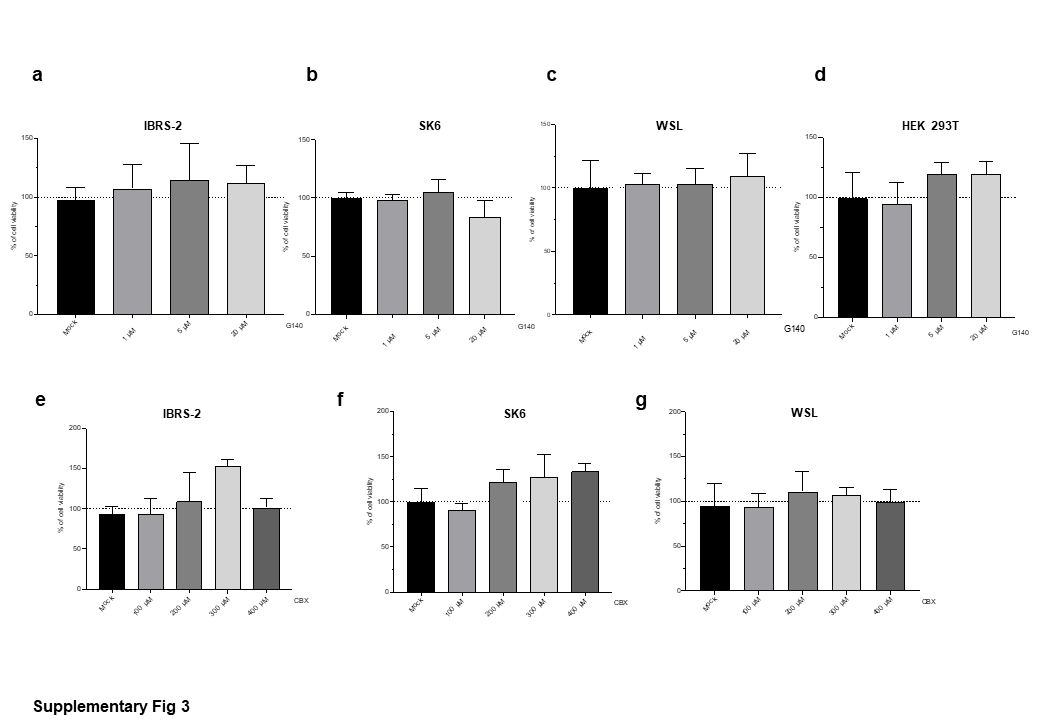

Supplement: Supplementary file 3 — Supplementary file3 (TIF 82 KB) [file 18_2024_5190_MOESM3_ESM.tif]

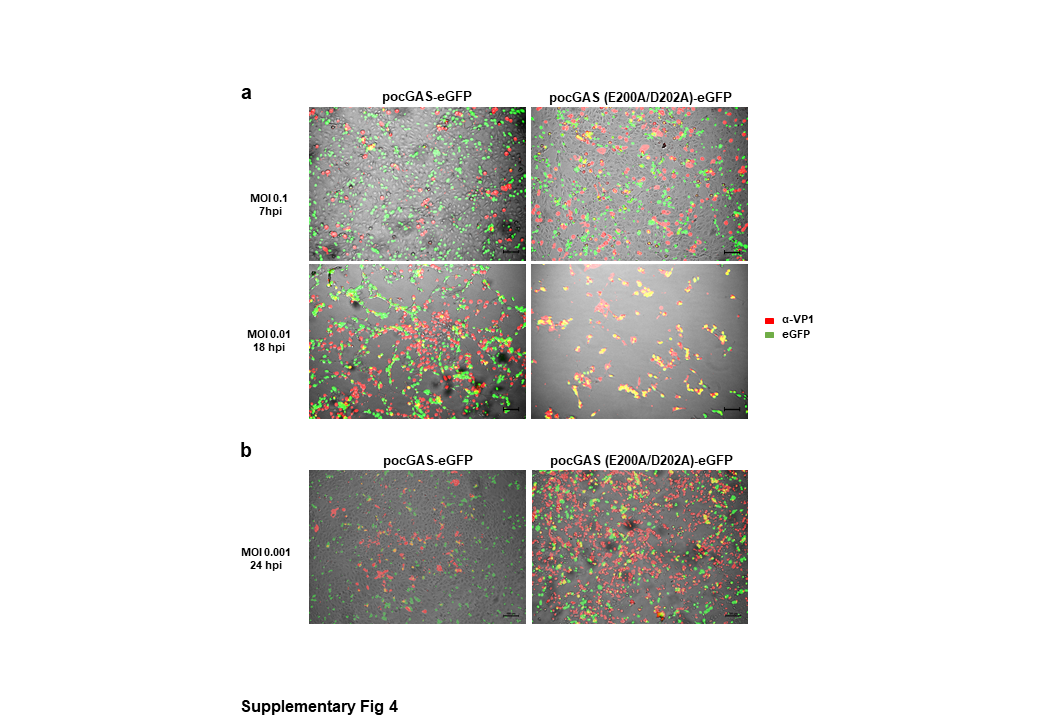

Supplement: Supplementary file 4 — Supplementary file4 (TIF 528 KB) [file 18_2024_5190_MOESM4_ESM.tif]

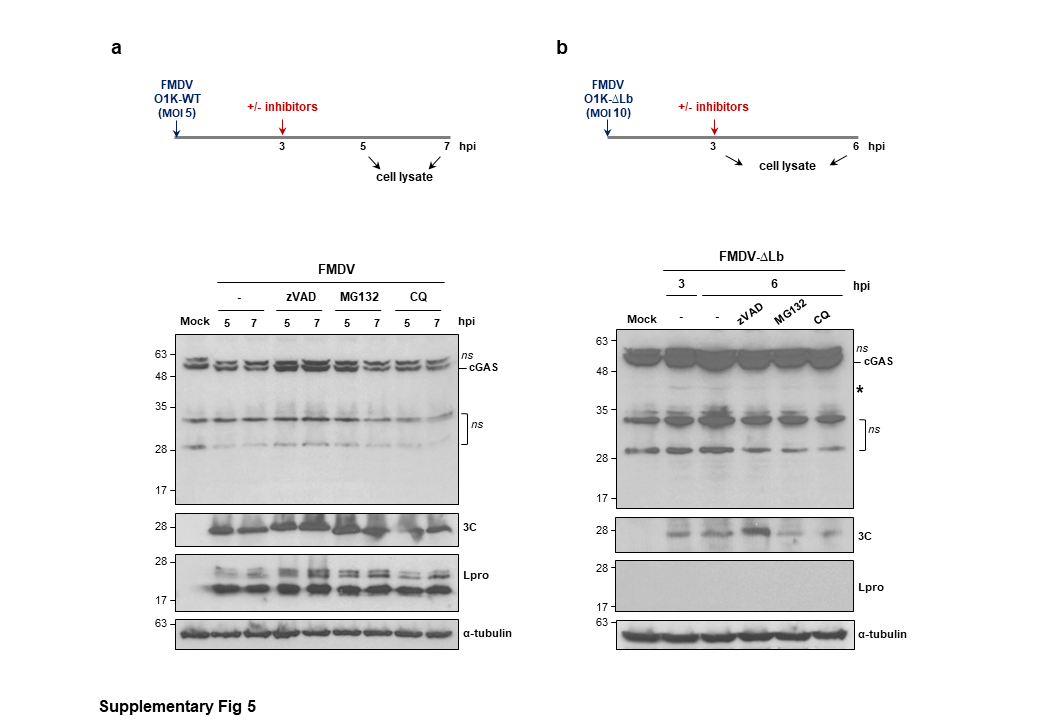

Supplement: Supplementary file 5 — Supplementary file5 (TIF 197 KB) [file 18_2024_5190_MOESM5_ESM.tif]
