# Supplementary material for: The antiviral response triggered by the cGAS/STING pathway is subverted by the foot-and-mouth disease virus proteases
Source: Cell Mol Life Sci. 2024 Mar 20;81(1):148. doi: 10.1007/s00018-024-05190-7 (PMC10954996; doi:10.1007/s00018-024-05190-7)

Fig 1a

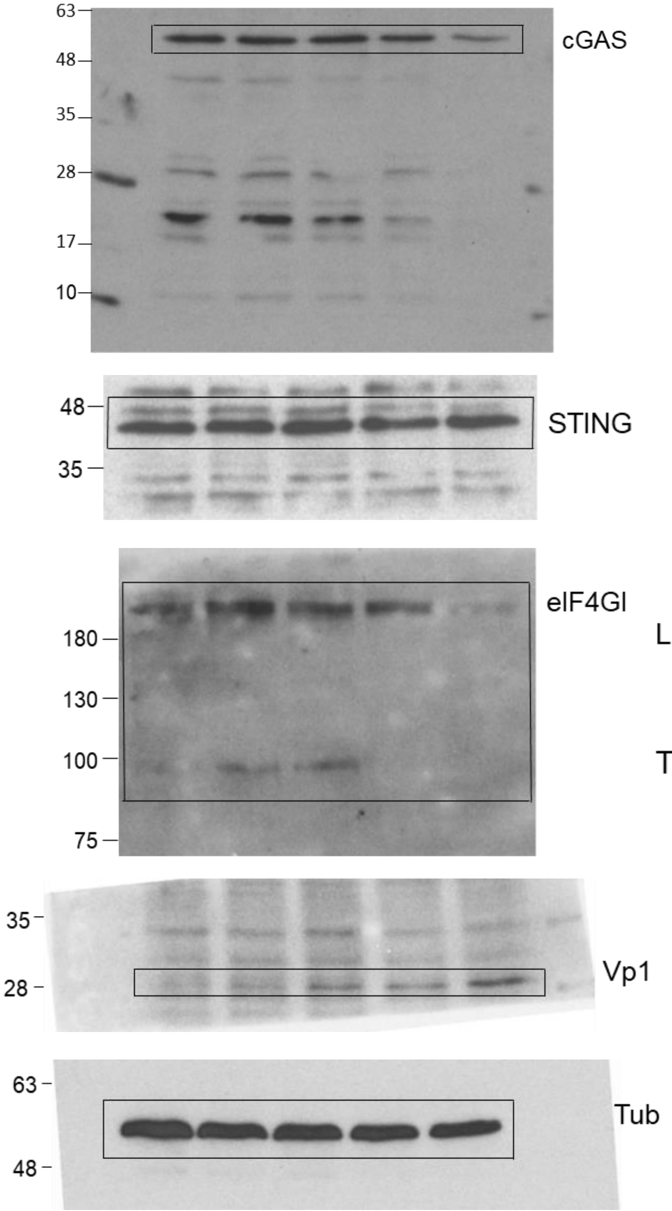

Fig 1b

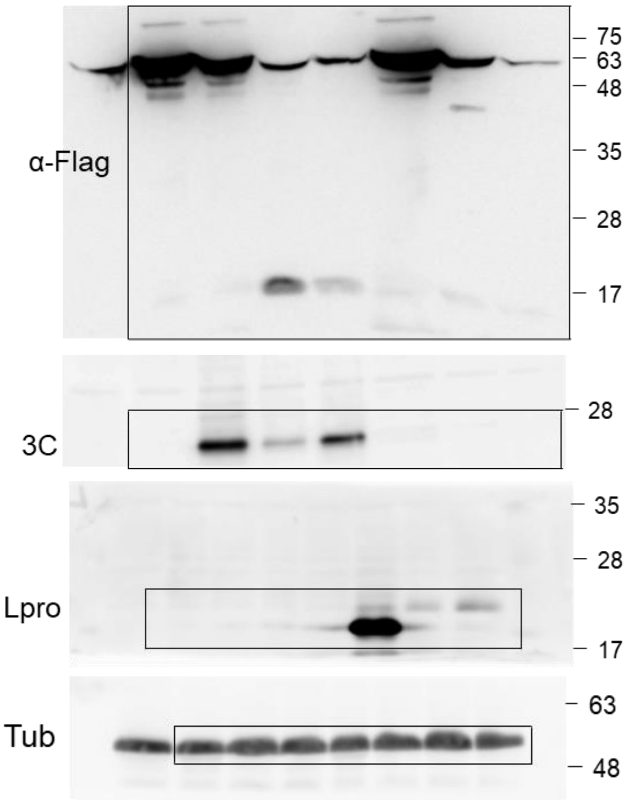

Fig 1c

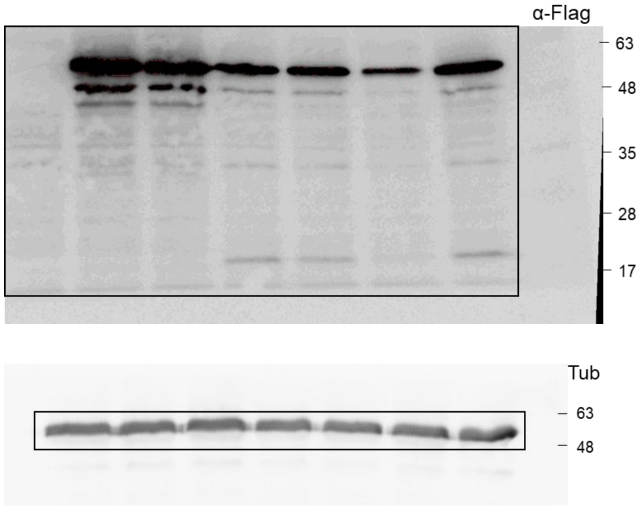

Fig 1d

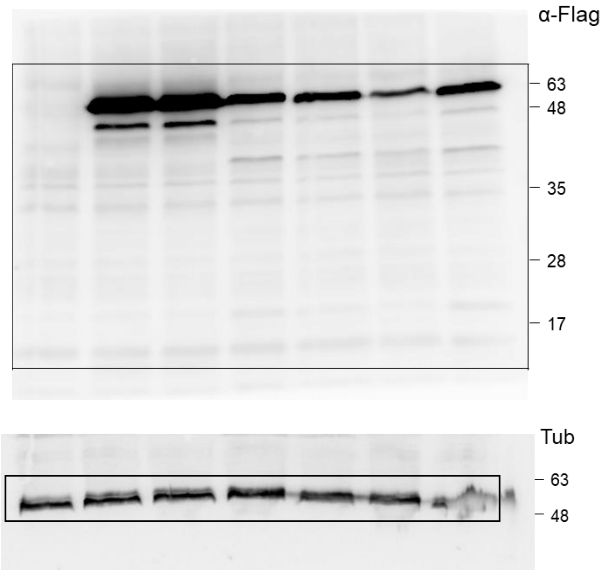

Fig 2a

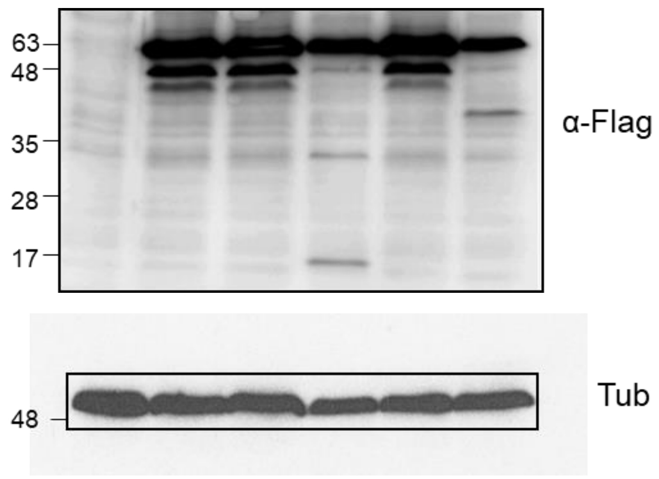

Fig 2b

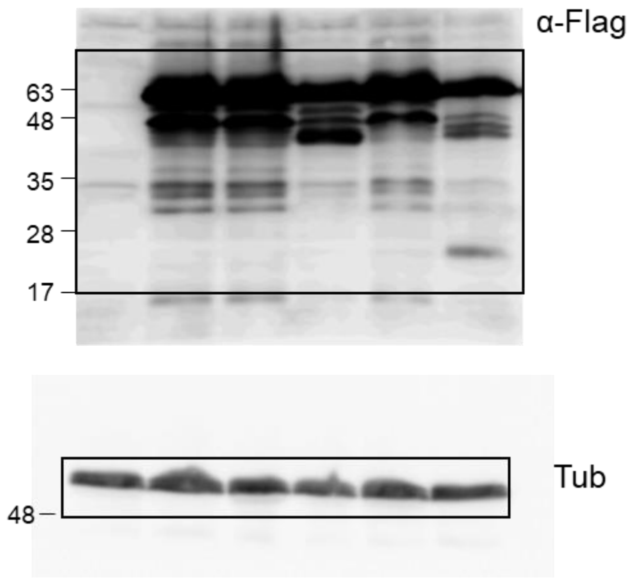

Fig 2c

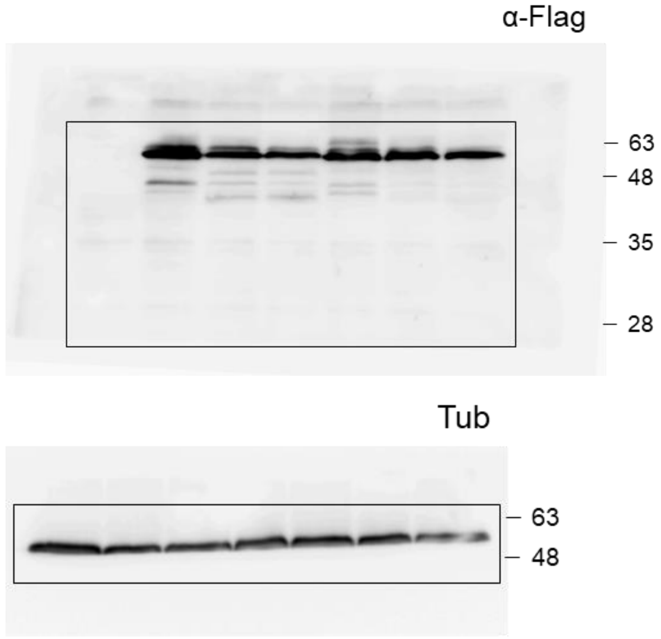

Fig 2d

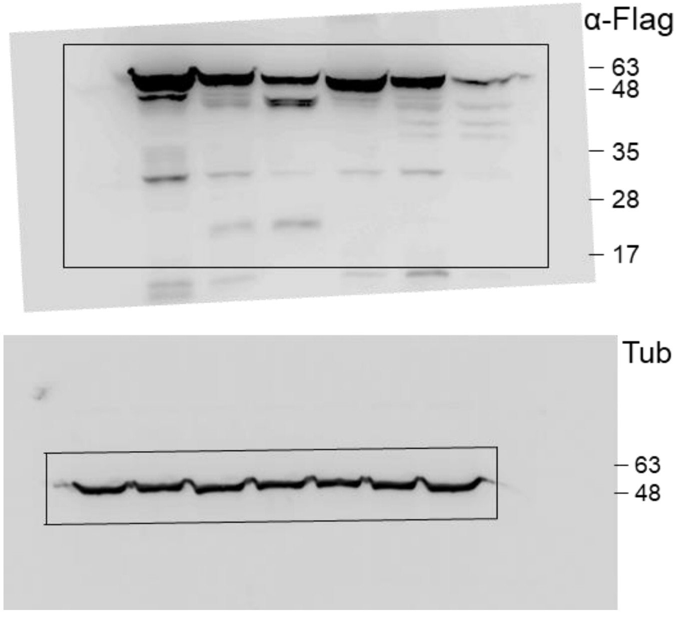

Fig 2f

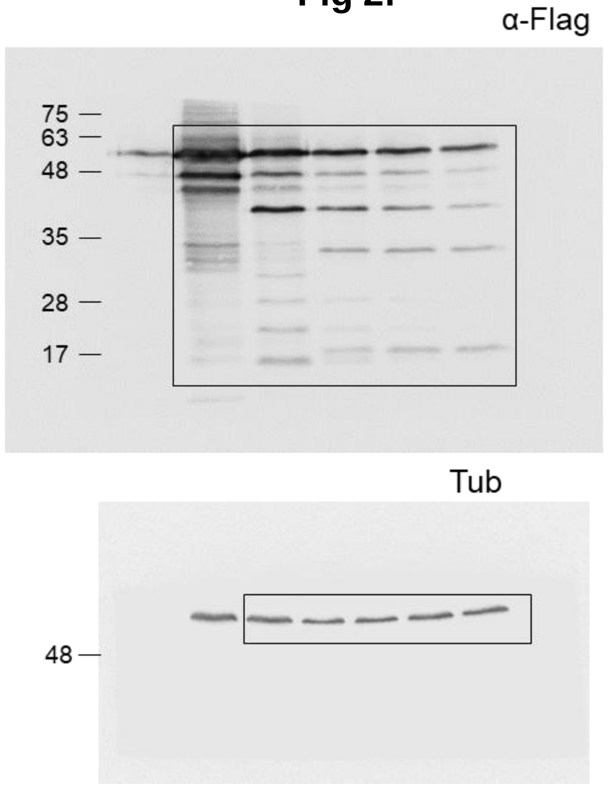

Fig 6a

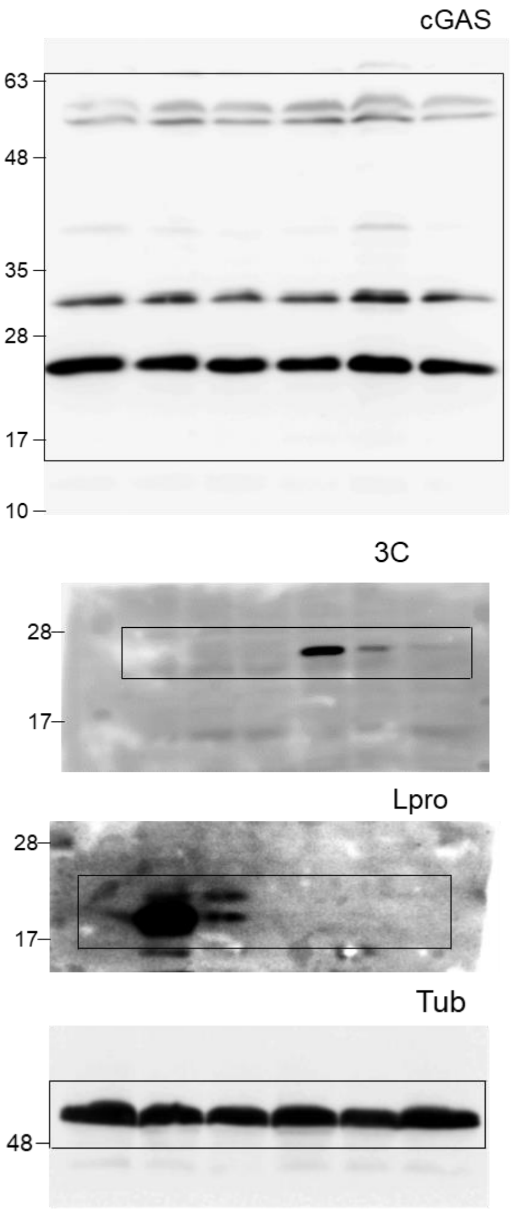
cGAS
63
48
35
28
17
10
3C
28
17
Lpro
28
17
Tub
48

Fig 6b

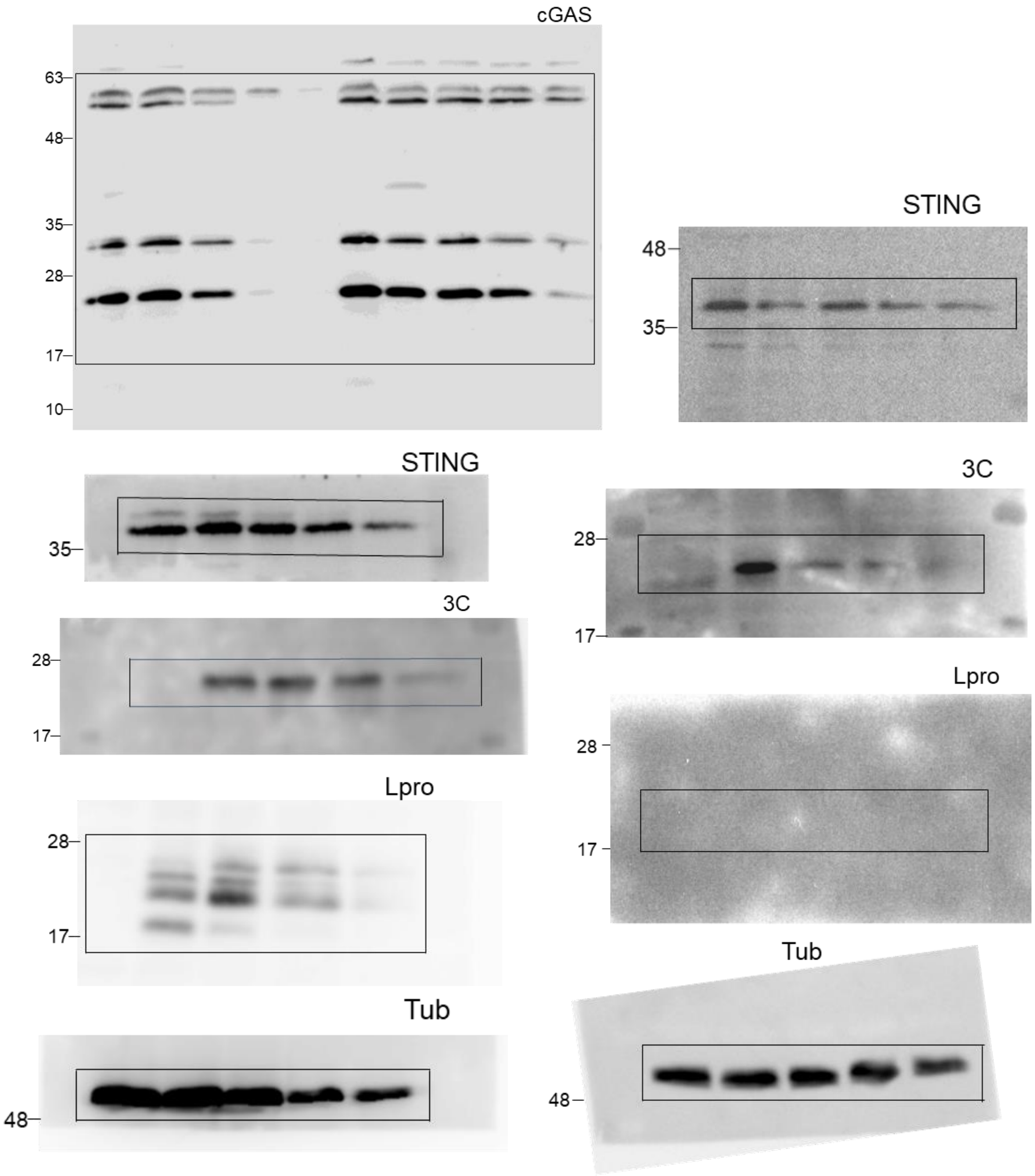
cGAS
63
48
35
28
17
10
STING
35
3C
28
17
Lpro
28
17
Tub
48
STING
48
35
3C
28
17
Lpro
28
17
Tub
48

Supplementary Fig 1a

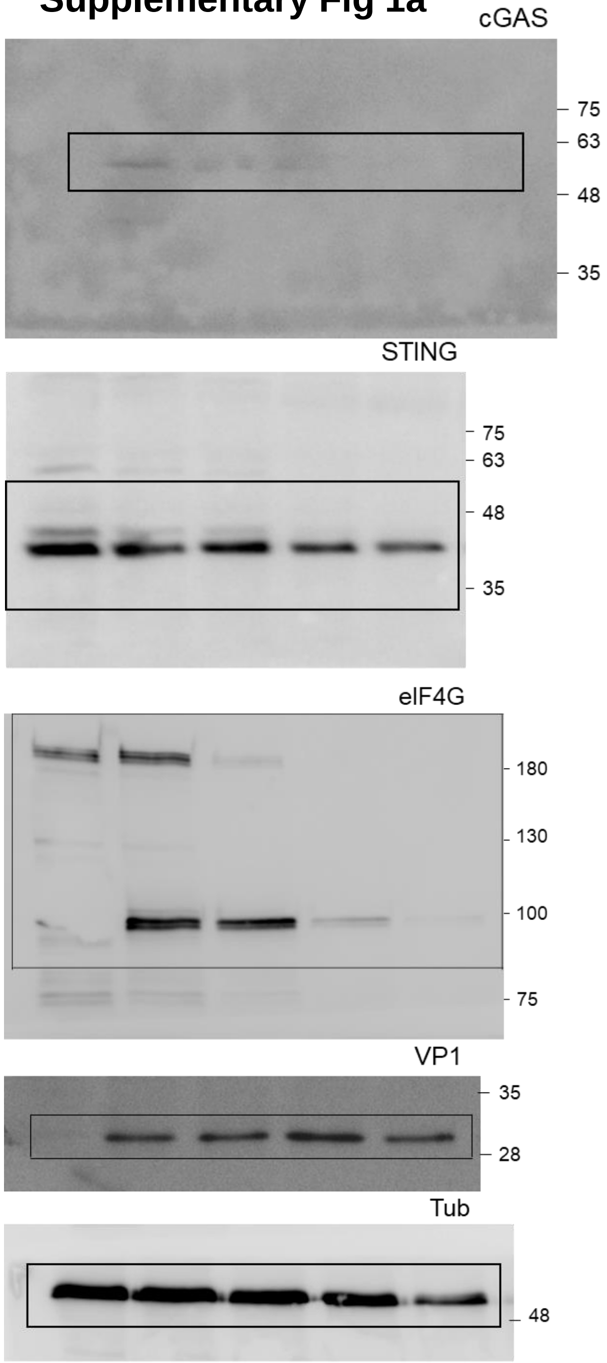

Supplementary Fig 1b

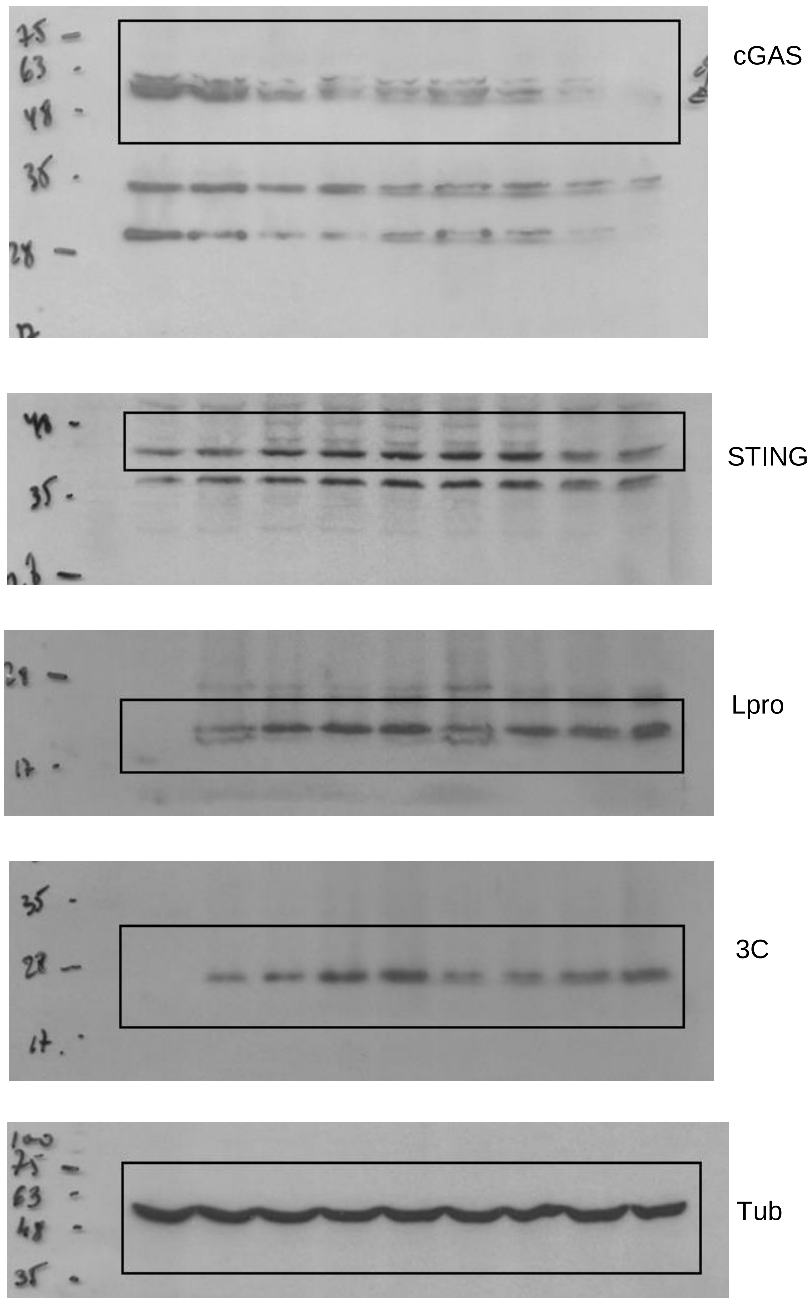

Supplementary Fig 2a

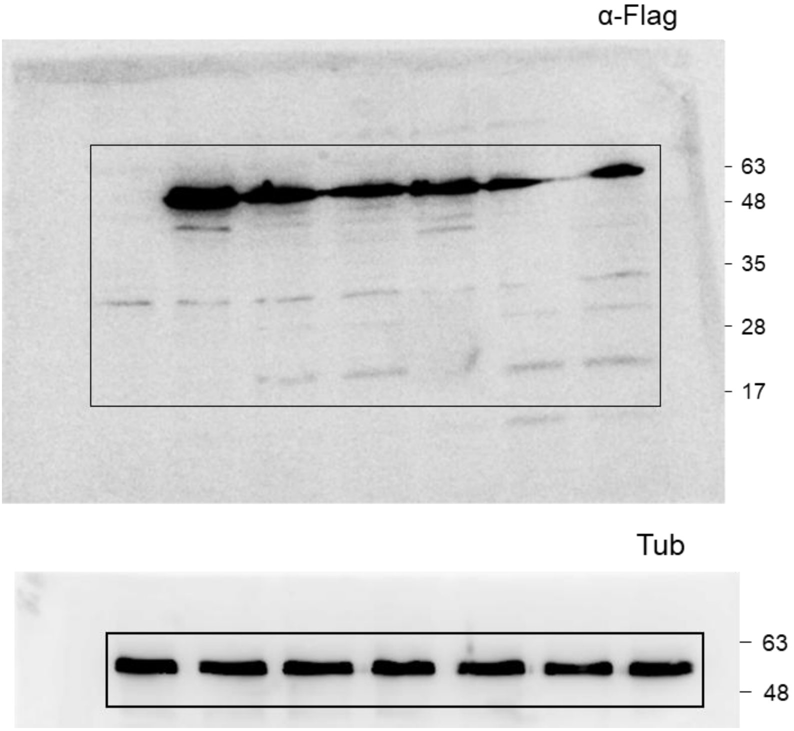

Supplementary Fig 2b

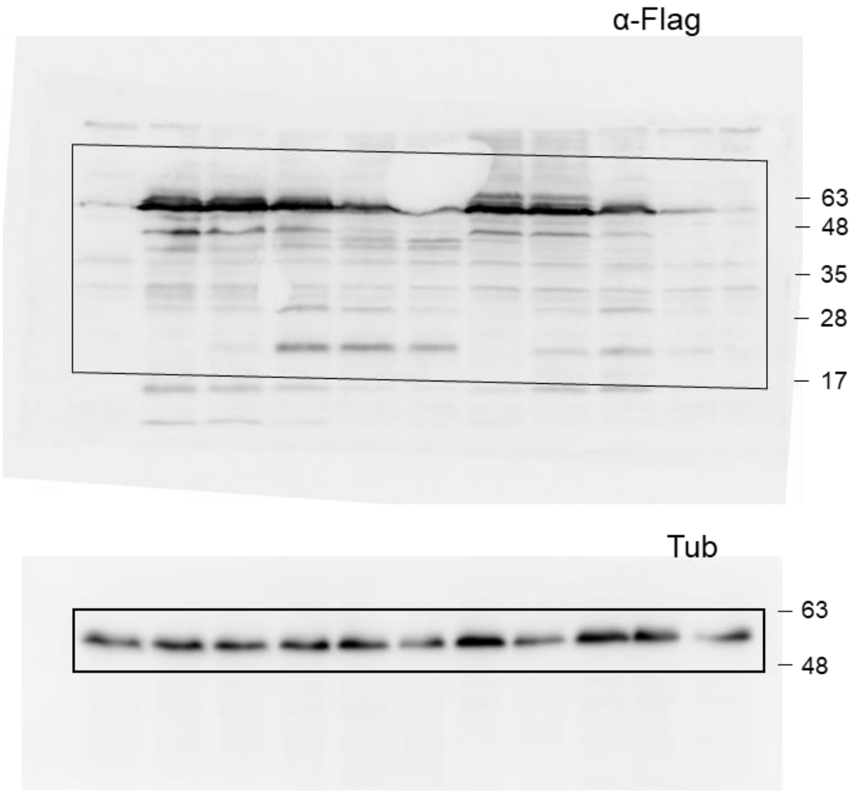

Supplementary Fig 5a

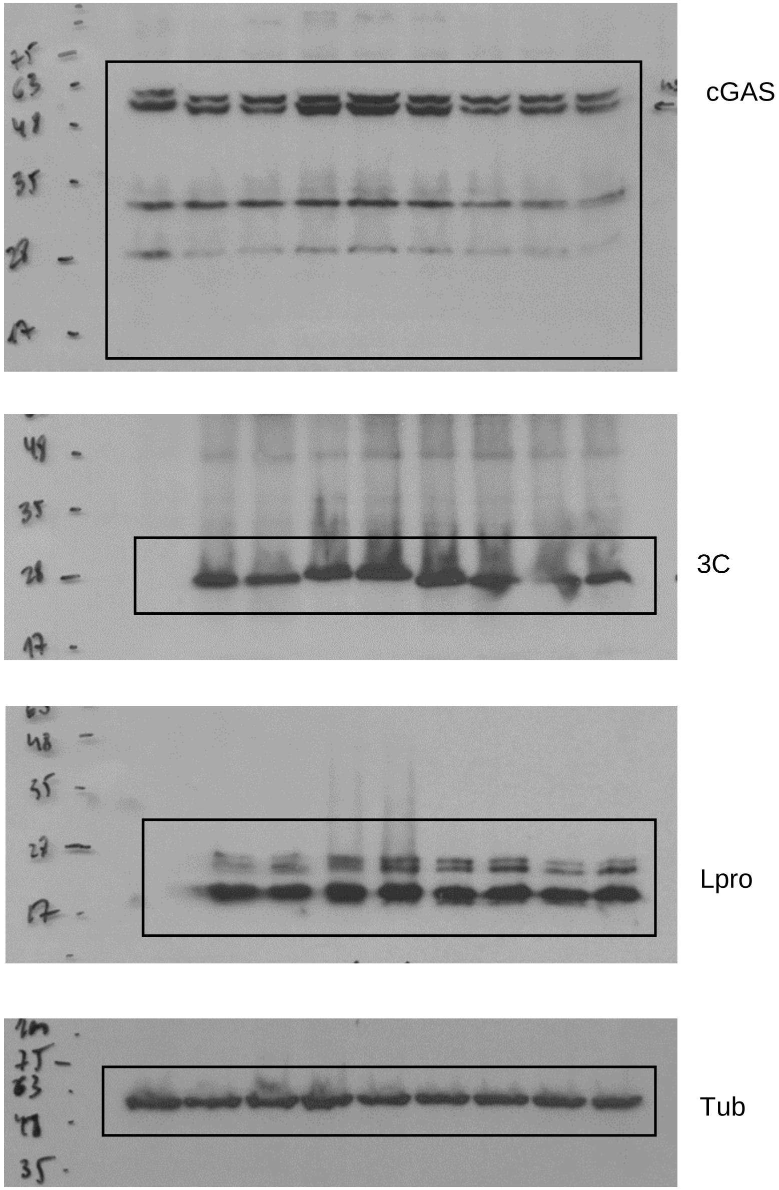

Supplementary Fig 5b

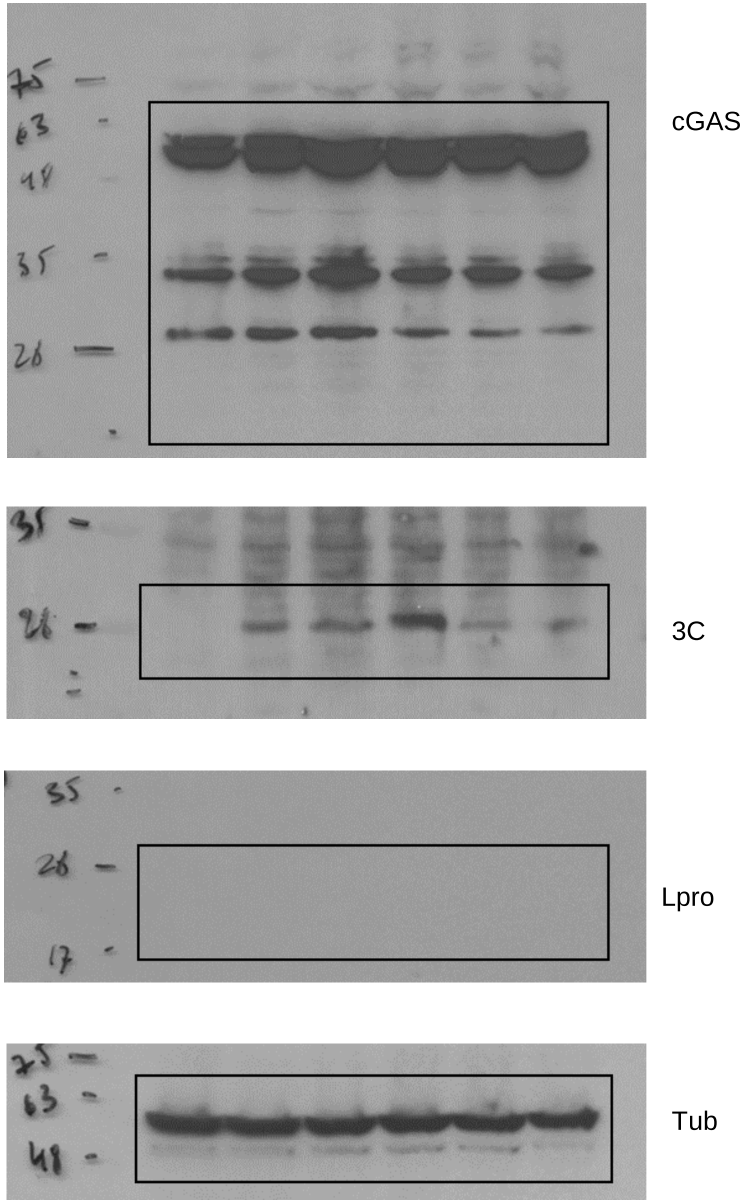

Supplement: Supplementary file 6 — Supplementary file6 (PDF 823 KB) [file 18_2024_5190_MOESM6_ESM.pdf]
